# A comprehensive review and meta-analysis on the safety and efficacy of esketamine for emerging delirium in elderly patients

**DOI:** 10.3389/fmed.2026.1752055

**Published:** 2026-02-06

**Authors:** Yanping Lu, Xuewei Wang, Henjing Zou, Cuiyuan Huang, Guangyi Lai, Shan Ou

**Affiliations:** 1 Department of Anesthesiology, Chengdu Intergrated TCM and Western Medicine Hospital, Chengdu, China; 2 Department of Emergency, Chengdu Second People's Hospital, Chengdu, China

**Keywords:** elderly, esketamine, meta-analysis, postoperative delirium, randomized controlled trials

## Abstract

**Background:**

Postoperative delirium (POD), a common complication in older surgical patients, has an incidence between 20 and 50%, highlighting the importance of effective intervention methods. Preventive methods available so far mainly focus on the reduction of well-known perioperative risks to prevent POD onset. Esketamine, a newly added therapeutic agent, has also exhibited the potential for POD incidence reduction and the improvement of patient outcomes. Previous meta-analyses and systematic reviews primarily focused on general adult patient groups; thus, the purpose of the current meta-analysis was specifically to examine the efficacy and safety outcomes of esketamine administration in surgical operations among elderly patients.

**Methods:**

This study was registered with PROSPERO (CRD420251151647). A literature search was conducted in the Cochrane Library, PubMed, Embase, Ovid, Scopus, and Web of Science databases until 1 October 2025. Eligible studies were English-language randomized controlled trials (RCTs) involving surgical patients aged ≥60 and reporting esketamine-related POD outcomes. Two reviewers independently selected studies and extracted data. The study quality was assessed using the Cochrane risk of bias (RoB) 2 tool. Data were pooled using fixed- or random-effects models and reported as risk ratios (RRs) or standardized mean differences (SMDs) with 95% CI. Heterogeneity was evaluated using I2 statistics, and publication bias was assessed using funnel plots and Egger’s test.

**Results:**

A total of 13 RCTs involving 1,581 elderly surgical patients were analyzed. Esketamine significantly reduced the incidence of POD (RR = 0.58, 95% CI: 0.43–0.79), postoperative nausea and vomiting (RR = 0.57, 95% CI: 0.44–0.74), perioperative hypotension (RR = 0.47, 95% CI: 0.27–0.81), and opioid consumption during anesthesia induction (SMD = −0.43, 95% CI: −0.59 to −0.26). However, it increased dizziness (RR = 1.29, 95% CI: 1.03–1.62) and perioperative hypertension (RR = 2.40, 95% CI: 1.16–4.97). No significant differences were found in pain scales [visual analog scale (VAS) and Numerical Rating Scale (NRS)], cognitive function [Mini-Mental State Examination (MMSE)], or psychological events (hallucinations and nightmares).

**Conclusion:**

Although perioperative esketamine exhibits benefits across multiple recovery outcomes, particularly in reducing intraoperative opioid consumption during induction, highlighting its potential for optimizing analgesic strategies, caution is warranted regarding the elevated risk of perioperative hypertension in elderly patients. The substantial heterogeneity observed in certain outcomes necessitates further validation. Therefore, current evidence does not yet support its routine clinical application.

**Systematic review registration:**

https://www.crd.york.ac.uk/PROSPERO/view/CRD420251151647, identifier CRD420251151647.

## 1 Introduction

POD is a serious postoperative complication among the elderly, characterized by disturbances in cognitive function, attention, and consciousness. It is strongly correlated with high morbidity, mortality, longer hospital stays, elevated medical costs, and decreased cognitive and physical functions (1). In contrast with a typical general surgical patient cohort, where the incidence of POD ranges from 2.5 to 3% (2, 3), elderly patients have higher rates, approximately 20 to 45% (4). In addition, up to 20–50% of older adults develop postoperative cognitive dysfunction 3 months after undergoing cardiac surgery (4). Due to its high prevalence and poor outcomes, effective management and objective assessment of POD are critically important, with evidence supporting the reduction of perioperative risks as an important method of prevention (5).

Esketamine, the S-enantiomer derivative of ketamine, was granted regulatory approval by the European Medicines Agency (EMA) and the US Food and Drug Administration (FDA) in 2019 (6). It gained interest as an emergent POD (postoperative delirium) therapeutic intervention because its N-methyl-D-aspartate (NMDA) receptors have superior affinity and strong, non-competitive antagonist activity (7, 8). Its increased receptor interaction allows esketamine to produce strong anesthetic and analgesic effects at lower doses than racemic ketamine, thereby reducing psychotomimetic symptoms and adverse effects (8, 9). Esketamine also shows increased clearance and a favorable safety profile (8). Owing to these properties, it is widely used in a variety of patient populations, such as pediatric, obstetric, adult, and geriatric patients. Its clinical applications include treatment-resistant depression, sedation and analgesia in perioperative settings, anxiety reduction, potential prevention of POD, and enhancement of postoperative recovery quality (10–14).

Given the favorable pharmacological profile of esketamine and the high clinical priority of POD in older surgical patients, the present meta-analysis aimed to systematically evaluate the preventive efficacy and safety of esketamine for POD prevention specifically in elderly surgical patients. Notably, this review focuses solely on the prevention (not the treatment) of POD. The use of esketamine for other indications, such as depression or analgesia unrelated to POD prevention, is not addressed, and comparisons with other pharmacological agents for POD prevention are not included. Furthermore, the analysis is limited to elderly patients; thus, the findings might not be generalizable to younger cohorts. The results are expected to provide evidence-based insights to guide clinical decision-making in the context of POD prevention.

## 2 Methods

This meta-analysis was performed following the Preferred Reporting Items for Systematic Reviews and Meta-Analyses (PRISMA) guidelines (15). The protocol was prospectively registered in the International Prospective Register of Systematic Reviews (PROSPERO) (16), available at https://www.crd.york.ac.uk/PROSPERO/view/CRD420251151647 (registration number: CRD420251151647).

### 2.1 Literature search and screening

Various electronic databases, including PubMed, EMBASE, the Cochrane Library, Web of Science, Scopus, and Ovid, were systematically searched from their inception to 1 October 2025. The search strategy used Medical Subject Headings (MeSH) and relevant free-text terms aligned with the study objectives, without language restrictions. Combinations of the following search terms were used: “Emergence Delirium,” “Esketamine,” “Ketamine,” “Aged,” and “Randomized Controlled Trial.” The search was conducted in English. Additionally, unpublished and ongoing clinical trials were identified via searches of ClinicalTrials.gov and the World Health Organization (WHO) International Clinical Trials Registry Platform (ICTRP).

**Figure 1:**
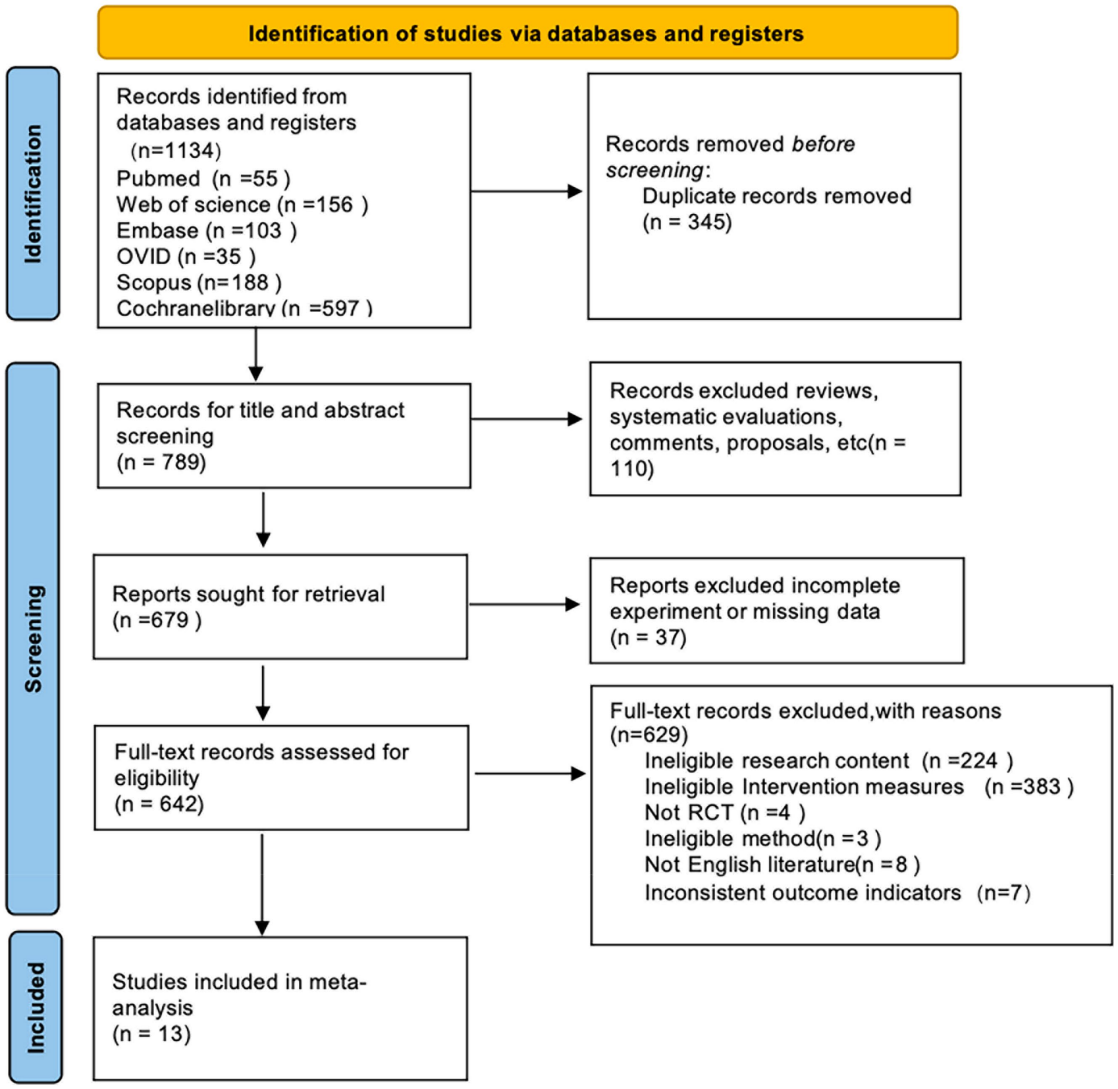
Flow diagram of the study selection process.

Two reviewers independently carried out database searches and conducted initial screening of the assembled studies by examining titles and abstracts. Any differences while screening were resolved unanimously through mutual discussion until complete agreement in the duo. Then, full texts were thoroughly assessed in accordance with previously set eligibility criteria (Figure 1).

### 2.2 Inclusion and exclusion criteria

Included studies were those with the following characteristics: (1) RCTs; (2) articles published in English; (3) inclusion of older patients aged ≥60 years, with no restrictions regarding sex, ethnic background, or duration of follow-up; (4) assessment of surgical procedures; and (5) reporting quantifiable outcomes regarding the effects of esketamine on POD. Studies were excluded if they were reviews, retrospective or observational studies, editorials, conference abstracts, duplicate publications, or contained insufficient data for analysis. Discrepancies in study selection were resolved through discussion with a third reviewer.

### 2.3 Data extraction

Two independent researchers systematically extracted data using a standardized spreadsheet developed in Microsoft Excel, which was subsequently imported into STATA version 15.0 for statistical analysis. Extracted information included study characteristics (e.g., authorship, year of publication, and study design), baseline patient characteristics, anesthetic techniques, esketamine dosage regimens and routes of administration, and predefined primary and secondary endpoints. The primary outcomes included the incidence of POD, postoperative pain intensity (assessed using VAS and/or NRS scales), Mini-Mental State Examination (MMSE) scores, and cumulative remifentanil consumption. The secondary outcomes comprised perioperative hemodynamic changes (hypotension and hypertension), quality of recovery (QoR-15) scores, and adverse events, including postoperative dizziness, hallucinations, nightmares, nausea, and vomiting. When essential data were missing, the corresponding authors were contacted to obtain additional information. If a trial reported outcomes as pre- and post-intervention means with standard deviations (SDs), mean differences and corresponding SDs were recalculated according to methods recommended by the Cochrane Handbook for Systematic Reviews of Interventions. For studies reporting data as medians and interquartile ranges, these values were converted to means and SDs according to Wan et al.’s approach (17). For the primary outcome (POD), in addition to reporting the relative risk (RR), clinically intuitive absolute effect measures were also provided. First, a single-rate meta-analysis was conducted on control group event rates to estimate the pooled baseline risk (control event rate, CER). Subsequently, the absolute risk reduction (ARR) and the number needed to treat (NNT) were calculated using the formulas ARR = CER × (1 − RR) and NNT = 1 / ARR, respectively, together with their 95% CI.

### 2.4 Risk of bias and certainty of evidence assessment

For trials providing repeated postoperative measurements, priority was given to data recorded closest to 24 h post-surgery. Two experienced assessors independently determined methodological robustness and bias risks for each included trial using the Cochrane Collaboration’s revised risk of bias assessment tool (RoB 2) via Review Manager software version 5.4 (18). This assessment included five specific domains: (1) adequacy of the randomization process, (2) deviations from intended interventions, (3) completeness of outcome data, (4) accuracy of outcome measurement, and (5) risk of selective reporting. Each domain was rated as “low risk” (low risk across all domains), “high risk” (high risk in at least one domain or some concerns in multiple domains), or “some concerns” (some concerns in at least one domain but no high risk in any domain). Discrepancies between reviewers were resolved by consultation with a third reviewer to achieve consensus.

### 2.5 Statistical analysis

STATA software (version 15.0) was used for all statistical analyses. The primary outcomes examined in the meta-analysis included the following: (a) incidence of POD; (b) postoperative pain intensity scores and outcomes on the Mini-Mental State Examination (MMSE); (c) remifentanil consumption; and (d) adverse effects due to therapy.

For binary data, aggregated findings were expressed using risk ratios (RRs) with their respective 95% confidence intervals (CIs). In contrast, continuous variables were summarized by calculating mean differences (MD) or, where outcome measures varied among trials, standardized mean differences (SMD), both reported alongside 95% CI. Study heterogeneity was quantified via the I^2^ index, categorized as negligible (≤25%), mild (25–50%), intermediate (50–75%), or considerable (>75%). A fixed-effects model was used when the I^2^ statistic indicated mild or negligible heterogeneity (≤50%), whereas a random-effects approach was adopted when heterogeneity exceeded this threshold. In cases of significant variability, subgroup and sensitivity analyses were undertaken to explore possible sources of heterogeneity. Furthermore, funnel plots and Egger’s regression tests were used to qualitatively and quantitatively evaluate publication bias.

## 3 Results

### 3.1 Overview of the included studies

First, our electronic database search across six databases identified 1,134 potential articles. After removing duplicates, 789 original records remained. During title and abstract screening, 110 irrelevant studies were excluded from the analysis. Subsequently, another 37 studies were excluded due to incomplete or insufficient data. Of the 642 full-text articles assessed, a further 629 were excluded for the following reasons: unrelated research aims (224), incompatible interventions (383), non-randomized study types (4), methodological incompatibilities (3), publications in languages other than English (8), and mismatched outcome measurements (7). Ultimately, 13 randomized controlled trials (RCTs) (19–31) met all the inclusion criteria for the meta-analysis. The careful screening and selection process is illustrated in Figure 1.

All 13 included studies enrolled patients aged over 60 years. One trial used spinal anesthesia, while the others used general anesthesia. Regarding esketamine administration, four studies administered it only during induction, five during both induction and intraoperative maintenance, and two solely for postoperative analgesia. One study used esketamine during induction, intraoperative maintenance, and postoperative analgesia; another combined induction with postoperative analgesia. Regarding control groups, one study used dexmedetomidine, two used opioid-based regimens, and the remaining studies used normal saline. Table 1 summarizes the detailed characteristics of the included RCTs.

**Table 1:** Characteristics of the included studies.

First author	Year	Study design and sample size (exp/con)	Age (Exp/Con)	Sex	Anesthesia	Time point of intervention	Ways of esketamine injection	Dosing regimen	Outcome(s)
Hireno Guará Sobrinho	2012	RCT 56 19/17/20	67.05 ± 7.04 64.12 ± 8.90 66.65 ± 8.27	M + F	Spinal anesthesia	Induction	Single dose	0.25 mg/kg;0.5 mg/kg	①②④⑥⑨
Juan Li	2021	RCT 80 40/40	68.8 ± 3.6 69.2 ± 5.4	M + F	General anesthesia	Induction	Single dose	0.2 mg/kg	①⑥⑨
Jiamin Ma	2023	RCT 39 19/20	69.45 ± 5.21 70.55 ± 4.24	M + F	General anesthesia	Induction+interaoperatively	Single dose+continuous	0.25 mg/kg;0.125 mg/kg/h	①③⑦⑧⑩⑫
Zhang, Yuan	2024	RCT 426 213/213	65 to 85 years	M + F	General anesthesia	Induction	Continuous	0.2 mg/kg/h	①③④⑦⑧⑩⑫
Jing Liu	2024	RCT 60 30/30	≥ 65 years old	M + F	General anesthesia	Postoperative analgesia	Continuous	1 mg/kg	①③⑤
Chao-Bang Ma	2024	RCT 260 130/130	69 ± 5.4 68.6 ± 4.1	M + F	General anesthesia	Induction+interaoperatively+postoperative analgesia	Single dose+continuous	0.2 mg/kg;0.125 mg/kg/h;0.5 mg/kg	①③④⑥⑨⑫
Huang, Cuifang	2024	RCT 209 108/101	63 ± 2.96 63 ± 2.96	M + F	General anesthesia	Induction+postoperative analgesia	Single dose+continuous	0.5 mg/kg; 2 mg/kg	①③④⑥⑨⑪
Zhaojun Jing	2024	RCT 87 44/43	69.2 ± 6.22 71.47 ± 6.18	M + F	General anesthesia	Induction+interaoperatively	Single dose+continuous	0.25 mg/kg;0.1 mg/kg/h	①②④⑤⑥⑨⑪⑫
Cong-Li Zhang	2024	RCT 163 82/81	70 ± 3.70 71 ± 3.70	M + F	General anesthesia	Induction+interaoperatively	Single dose+continuous	0.25 mg/kg;0.125 mg/kg/h	①③④⑤⑥⑪⑫
Yongjian Lu	2024	RCT 94 47/47	70.05 ± 5.13 69.25 ± 5.21	M + F	General anesthesia	Induction	Single dose	0.5 mg/kg	①②④⑦⑩
Qiuping Ye	2024	RCT 121 61/60	73 ± 11.85 71 ± 10.37	M + F	General anesthesia	Induction+interaoperatively	Single dose+continuous	0.5 mg/kg,0.25 mg/kg/h	①②⑤⑦⑧
Yan Zhao	2025	RCT 112 57/55	70 ± 4 70 ± 5.1	M + F	General anesthesia	Postoperative analgesia	Continuous	0.72 mg/kg	①④⑤⑩⑪⑫
Xuandong Jia	2025	RCT 80 40/40	65 and 92 years	M + F	General anesthesia	Induction+interaoperatively	Single dose+continuous	0.3 mg/kg;0.3 mg/kg/h	①④⑦⑪

① Postoperative delirium (POD); ② visual analog scale (VAS); ③ Numerical Rating Scale (NRS); ④ dizziness; ⑤ postoperative nausea and vomiting; ⑥ hallucinations; ⑦ intraoperative hypotension; ⑧ intraoperative hypertension; ⑨ nightmares; ⑩ Mini-Mental State Examination (MMSE); ⑪ quality of recovery (QoR-15); ⑫ remifentanil.

M, male; F, female; RCT, randomized controlled trial; Exp, experimental group; Con, control group.

### 3.2 Quality assessment

**Figure 2:**
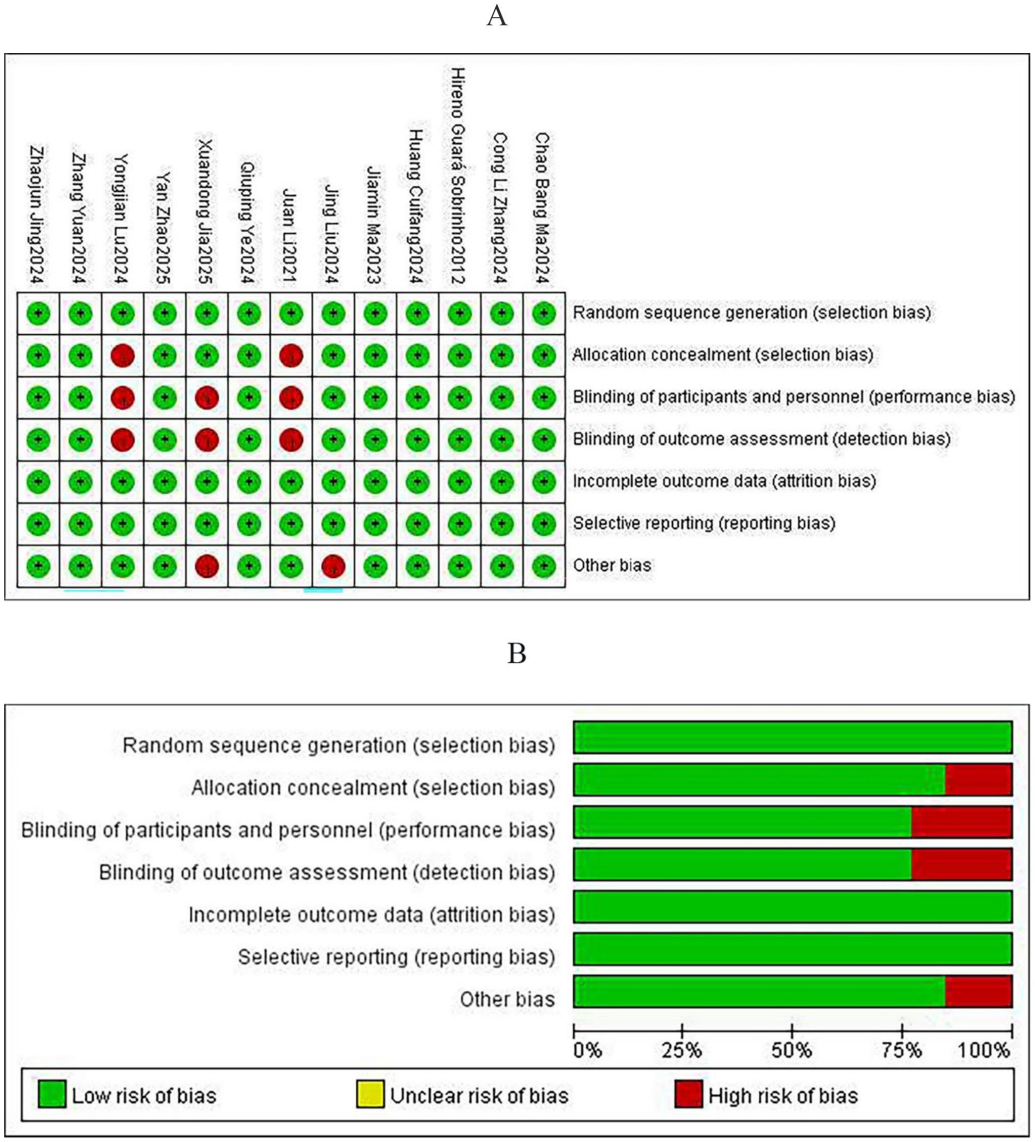
Risk of bias assessment: **(A)** Risk of bias for individual studies and **(B)** risk of bias summary.

The evaluation of bias risk for the 13 RCTs included was carried out using Review Manager (version 5.4). Among these studies, insufficient information regarding the generation of randomization sequences was noted in one trial, and another two trials provided inadequate descriptions of concealment methods for group allocation. Additionally, three studies did not sufficiently document details of blinding procedures for participants, while baseline characteristics were incompletely described in two other trials. Overall, the risk of bias was assessed as low in nine studies, while four studies were judged to have some concerns. Detailed summaries of the risk of bias assessments for individual trials and the overall quality evaluation of the included studies are presented in Figures 2A,B.

### 3.3 Meta-analyses of outcomes

#### 3.3.1 Primary outcomes

##### 3.3.1.1 POD

**Figure 3:**
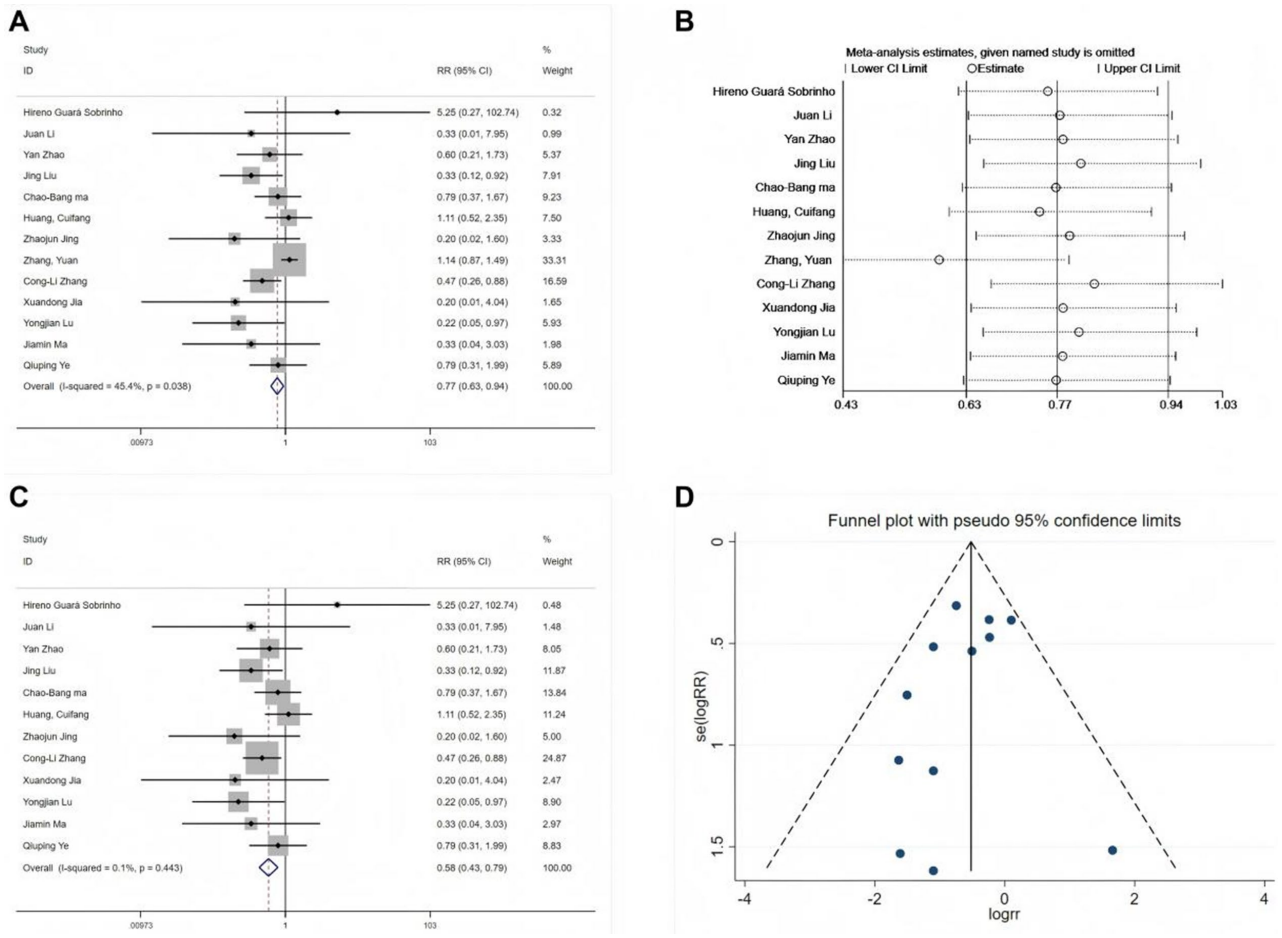
Forest plots and sensitivity analysis for the effect of esketamine on POD. **(A)** Initial fixed-effects meta-analysis of 13 studies, **(B)** sensitivity analysis, **(C)** final fixed-effects meta-analysis of 12 studies, and **(D)** funnel plot for publication bias assessment of the final model.

All 13 studies reported the incidence of POD (11, 19–22, 24–31). Initial heterogeneity analysis revealed moderate variation (I^2^ = 45.4%, *p* = 0.038; Figure 3A), indicating statistically significant heterogeneity. Sensitivity analysis (Figure 3B) identified the study by Zhang et al. (30) as a substantial source of heterogeneity, primarily due to the assessment of POD incidence based on morning versus afternoon surgeries. After excluding this study, reassessment of the remaining 12 studies indicated no significant heterogeneity (I^2^ = 0.1%, *p* = 0.443). A fixed-effects model was then used in the meta-analysis (RR = 0.58, 95% CI: 0.43–0.79, *p* < 0.001; Figure 3C), exhibiting a 42% reduction in POD risk following perioperative esketamine administration. Funnel plot analysis (Figure 3D) and Begg’s test (*p* > 0.05) indicated no significant publication bias. The pooled incidence of POD in the control group was 13% (95% CI: 11–16%). Based on this finding, the absolute risk reduction (ARR) was calculated as 0.063 (95% CI: 0.034–0.063), and the number needed to treat (NNT) was 16 (95% CI: 16–29). Thus, among elderly surgical patients with a baseline POD risk of approximately 13%, perioperative esketamine administration to approximately 16 patients would be required to prevent one case of POD.

Subgroup analyses were conducted based on clinically relevant indicators. Since only one study used neuraxial anesthesia and all others used general anesthesia, subgrouping by anesthesia type was not performed. Instead, analyses were stratified according to two dimensions: (1) esketamine administration during anesthesia induction and (2) its use for postoperative analgesia.

Subgroup analysis revealed that esketamine administration during induction significantly reduced POD risk (RR = 0.63, 95% CI: 0.44–0.91; 10 studies), whereas administration at non-induction time points did not reduce POD risk (RR = 0.69, 95% CI: 0.31–1.52; 3 studies). Meta-regression analysis confirmed that administration timing had no statistically significant impact (*p* = 0.405). Furthermore, a subgroup analysis based on postoperative analgesia use revealed no significant differences.

##### 3.3.1.2 Postoperative pain scores

**Figure 4:**
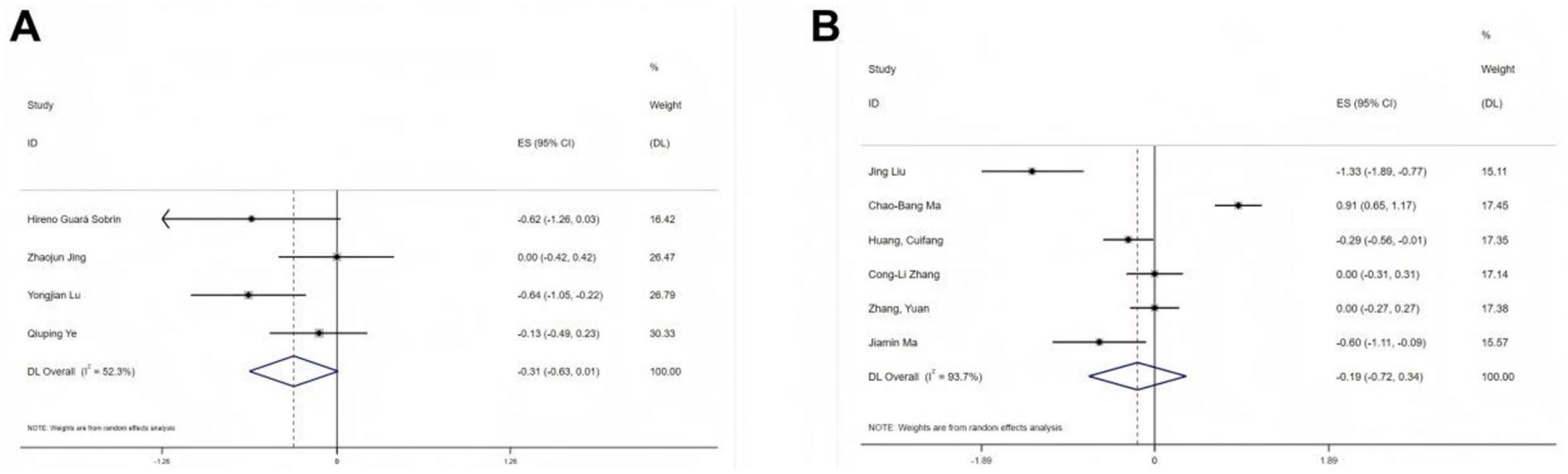
Forest plots illustrating the effect of esketamine on postoperative pain: **(A)** Visual analog scale (VAS; four studies, random-effects model) and **(B)** Numerical rating scale (NRS; seven studies, random-effects model).

Postoperative pain scores were reported in 10 studies using two different scales: VAS and NRS. Separate meta-analyses were conducted for each scale. Among the four studies (19, 22, 25, 28) using the VAS, substantial heterogeneity was observed (I^2^ = 52.3%, *p* < 0.1). Therefore, a random-effects model was used. Despite further investigation, the source of heterogeneity remained unclear. Comparing the esketamine group to the control group, no significant difference was found in postoperative pain (SMD = −0.31, 95% CI: −0.63- 0.01, Figure 4A). Similarly, six studies (20, 21, 24, 26, 29, 30) using the NRS scale also exhibited substantial heterogeneity (I^2^ = 93.7%, *p* < 0.1), and no specific source of heterogeneity was identified. The results from the random-effects model were consistent with the VAS findings (SMD = −0.19, 95% CI: −0.72 to 0.34, Figure 4B).

##### 3.3.1.3 MMSE scores

**Figure 5:**
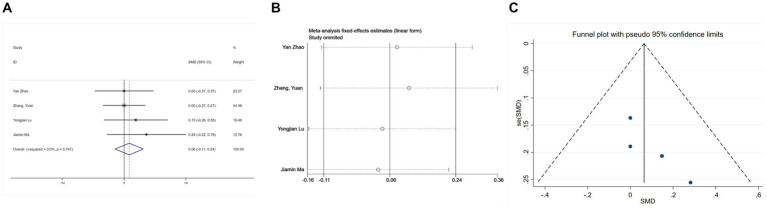
Forest plots and sensitivity analysis for the effect of esketamine on mental status: **(A)** Fixed-effects meta-analysis of four studies; **(B)** sensitivity analysis; and **(C)** funnel plot for publication bias assessment.

Four studies (25, 26, 30, 31) provided postoperative Mini-Mental State Examination (MMSE) data. Due to negligible heterogeneity (I^2^ = 0%, *p* = 0.747 > 0.1), a fixed-effects model was deemed appropriate. The combined analysis indicated no significant influence of esketamine on MMSE scores (SMD = 0.06, 95% CI: −0.11 to 0.24, *p* = 0.477, Figure 5A), suggesting that esketamine does not markedly alter postoperative cognitive function. A sensitivity analysis (Figure 5B) revealed no undue influence from any single study, confirming the stability of these findings. Furthermore, funnel plot inspection showed symmetry, and Begg’s test (*p* > 0.05, Figure 5C) revealed no publication bias.

##### 3.3.1.4 Remifentanil consumption

**Figure 6:**
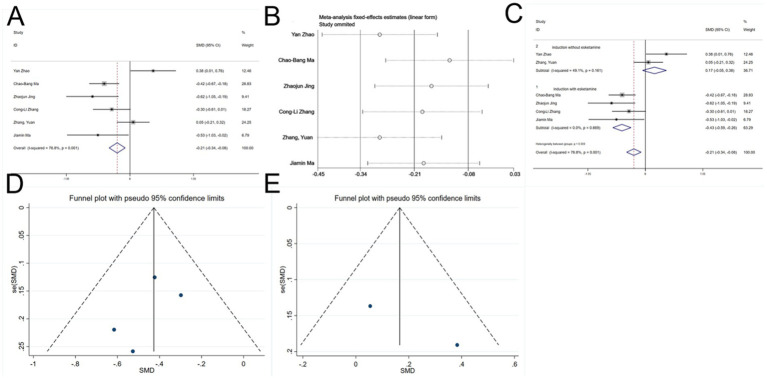
Forest plots and sensitivity analysis for the effect of esketamine on remifentanil consumption: **(A)** Initial fixed-effects meta-analysis of six studies; **(B)** sensitivity analysis; **(C)** subgroup analysis; **(D)** funnel plot for publication bias assessment (esketamine induction subgroup); and **(E)** funnel plot for publication bias assessment (no esketamine induction subgroup).

Six studies (22, 26, 27, 30, 31) reported total perioperative remifentanil consumption. Significant heterogeneity was detected (I^2^ = 76.8%, *p* < 0.1, Figure 6A). Variability in esketamine administration during induction was suspected as the cause. Sensitivity analysis (Figure 6B) clearly identified two subgroups based on the induction use of esketamine. A meta-regression analysis was then conducted (Table 2), using esketamine administration during induction as the independent variable, confirming its significant influence on the effect size (*p* < 0.05). Subsequently, a subgroup analysis was performed. To investigate the source of heterogeneity, a meta-regression analysis was performed with a single covariate indicating whether esketamine was administered during induction (coded as yes = 1, no = 0) to quantitatively evaluate its impact on the pooled effect size (remifentanil consumption).

**Table 2:** Meta-regression analysis.

ES	Exp (b)	Std. Err.	*T*	*P* > |t|	[95% Conf. Interval]	
Subgroup	1.808967	0.2527246	4.24	0.013	1.227364	2.666172
Cons	0.3607383	0.0730557	−5.03	0.007	0.2055881	0.6329749

ES, effect size; exp (b), exponentiated coefficient (odds ratio scale); Std. Err., standard error; subgroup, binary covariate (1 = esketamine used at induction, 0 = no esketamine at induction); cons, model intercept.

Among the four studies (22, 26, 27, 30) in which esketamine was administered at induction, no heterogeneity was observed. The results indicated that esketamine administered at induction significantly reduced intraoperative remifentanil consumption (SMD = −0.43, 95% CI: −0.59 - -0.26, *p* < 0.05, Figure 6C). In contrast, two studies ((30, 31)) without induction administration of esketamine showed no heterogeneity, and the results revealed no significant difference compared to controls (SMD = 0.17, 95% CI: −0.05 - 0.38, *p* > 0.05, Figure 6C). Thus, esketamine administration during induction reduced perioperative remifentanil use.

Funnel plots for both subgroups were essentially symmetrical (Figures 6D,E), and Begg’s tests (*p* > 0.05) indicated no publication bias.

#### 3.3.2 Secondary outcomes

##### 3.3.2.1 Nausea and vomiting, dizziness, hallucinations, and nightmares

**Figure 7:**
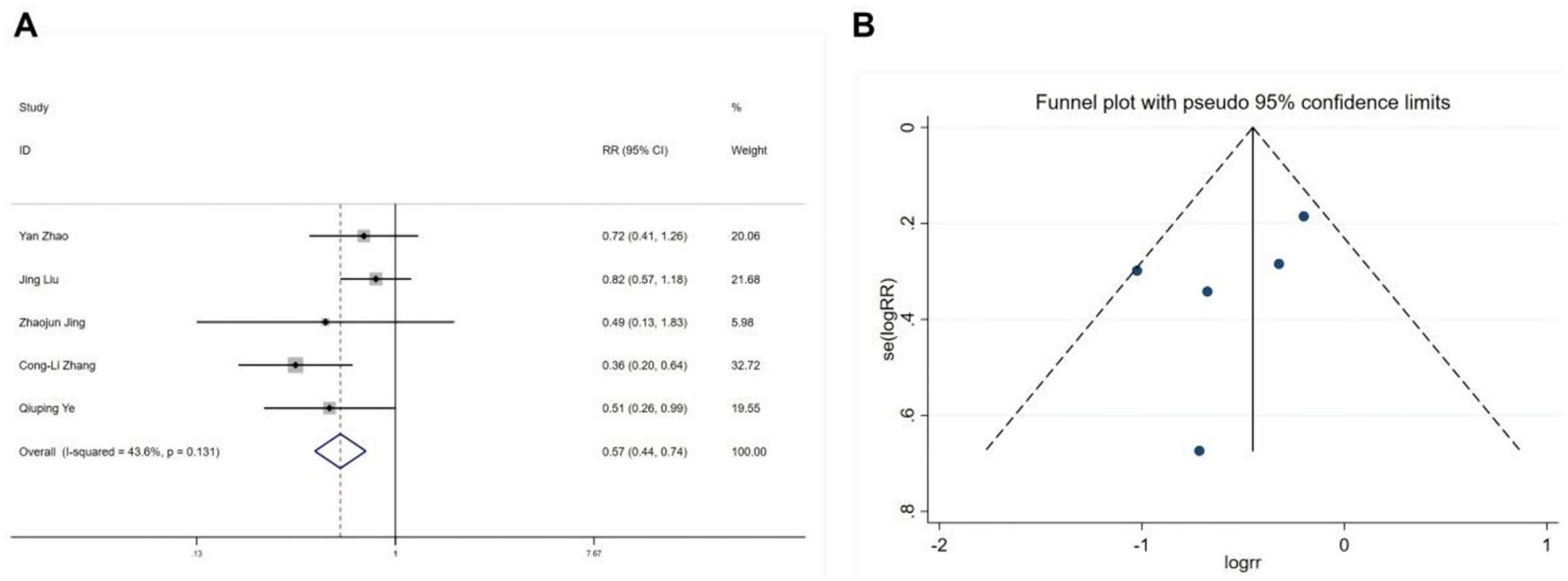
Forest plot and funnel plot for the effect of esketamine on postoperative nausea and vomiting. **(A)** Fixed-effects meta-analysis of five studies and **(B)** funnel plot assessing publication bias.

**Figure 8:**
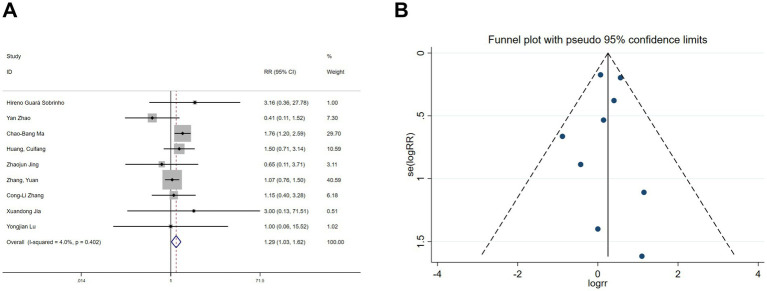
Forest plot and funnel plot for the effect of esketamine on dizziness. **(A)** Fixed-effects meta-analysis of nine studies and **(B)** funnel plot assessing publication bias.

Five studies (22, 28, 30, 31) reported postoperative nausea and vomiting, and nine studies (19–22, 25, 26, 30, 31) reported postoperative dizziness. Heterogeneity tests showed no significant heterogeneity (nausea and vomiting: I^2^ = 43.6%, *p* = 0.131; dizziness: I^2^ = 4%, *p* = 0.402). Fixed-effects model analyses indicated that esketamine significantly reduced postoperative nausea and vomiting (RR = 0.57, 95% CI: 0.44–0.74, *p* < 0.001, Figure 7A) but increased the incidence of dizziness (RR = 1.29, 95% CI: 1.03–1.62, *p* = 0.026, Figure 8A). Funnel plots (Figures 7B, 8B) and Begg’s bias tests confirmed that there was no publication bias.

**Figure 9:**
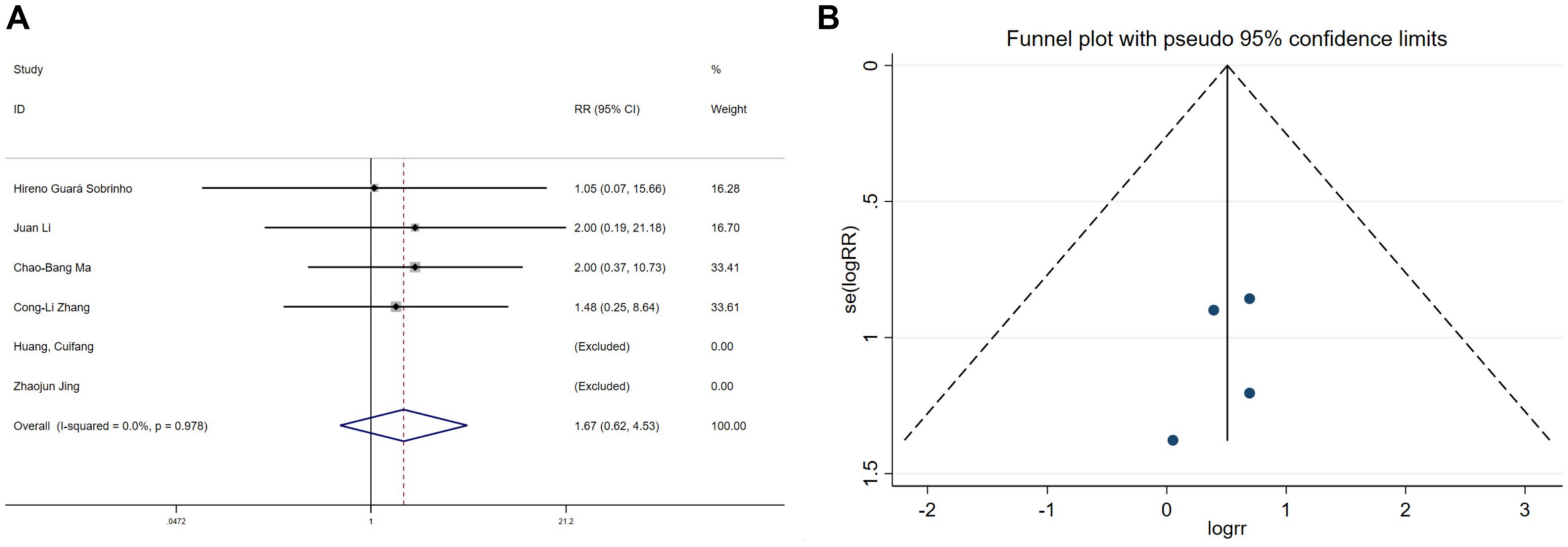
Forest plot and funnel plot for the effect of esketamine on hallucinations. **(A)** Fixed-effects meta-analysis of six studies and **(B)** funnel plot assessing publication bias.

**Figure 10:**
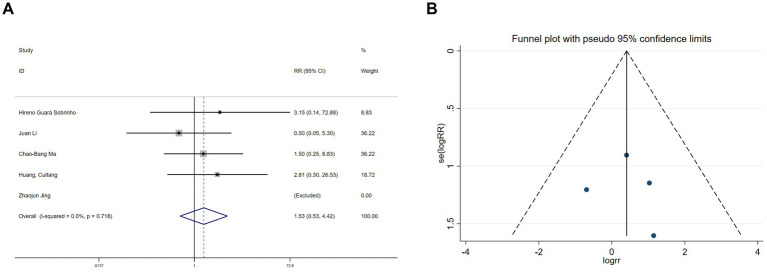
Forest plot and funnel plot for the effect of esketamine on nightmares. **(A)** Fixed-effects meta-analysis of five studies and **(B)** funnel plot assessing publication bias.

Hallucination data were extracted from six studies (11, 19, 20, 22, 26, 30) and nightmare data from five studies (11, 19, 20, 22, 26). Heterogeneity assessments indicated minimal variability among the trials (hallucinations: I^2^ = 0%, *p* = 0.978; nightmares: I^2^ = 0%, *p* = 0.718). Using a fixed-effects model, the meta-analysis exhibited no significant benefit of esketamine in reducing hallucinations (RR = 1.67, 95% CI: 0.62–4.53, *p* = 0.313, Figure 9A) or nightmares (RR = 1.53, 95% CI: 0.53–4.42, *p* = 0.434, Figure 10A). Funnel plots (Figures 9B, 10B) and Begg’s bias tests further confirmed the absence of publication bias (hallucinations: *p* = 0.308; nightmares: *p* = 1).

##### 3.3.2.2 Qor-15 scores

**Figure 11:**
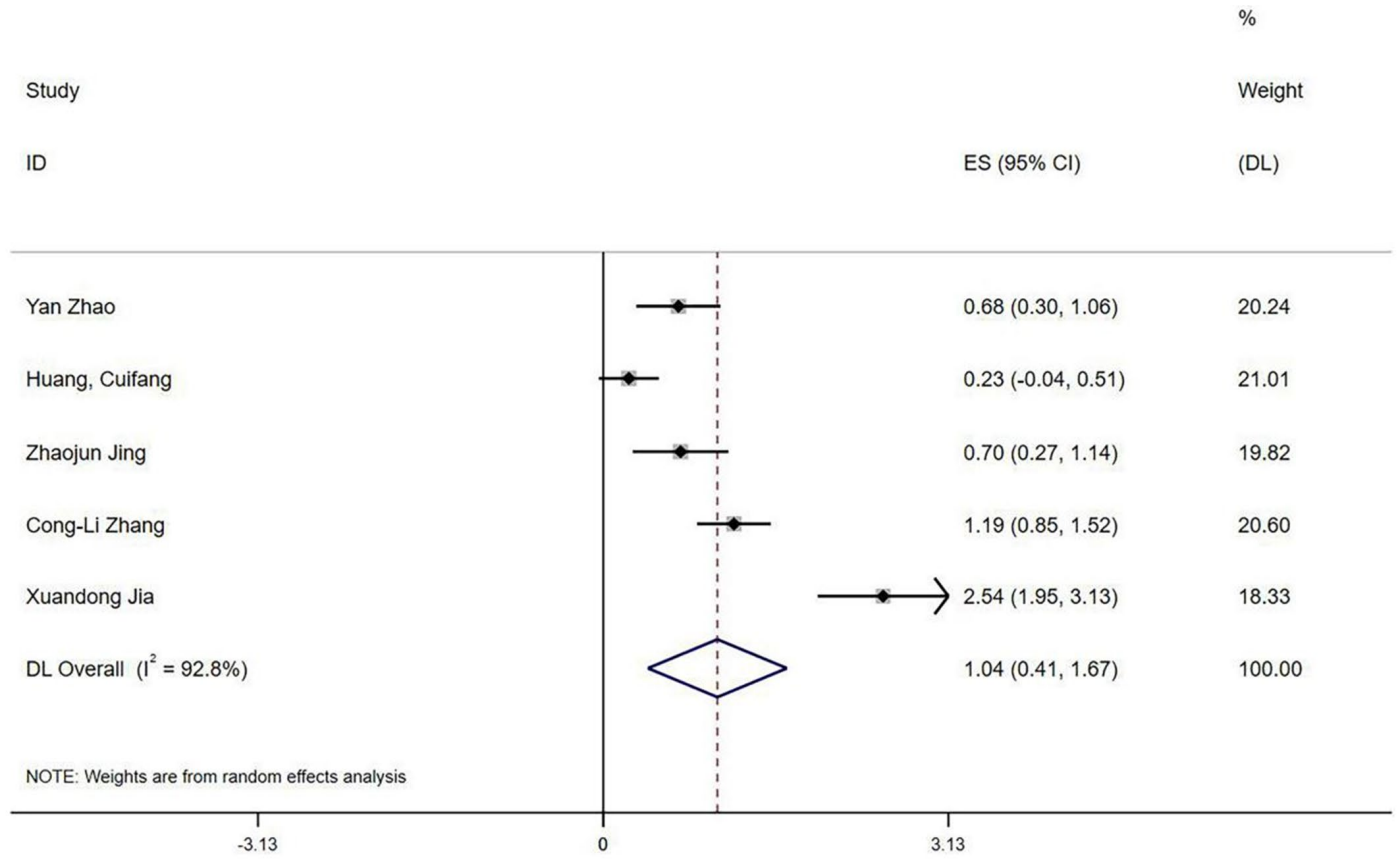
Forest plot of QoR-15 scores (random-effects model).

Five studies (20–22, 31) reported QoR-15 scores. Heterogeneity testing revealed significant variation (I^2^ = 92.8%, *p* < 0.1). Sensitivity analysis could not identify the source of heterogeneity. A random-effects model meta-analysis indicated that esketamine significantly improved postoperative recovery quality (SMD = 1.04, 95% CI: 0.41–1.67, Figure 11).

##### 3.3.2.3 Abnormal blood pressure

###### 3.3.2.3.1 Intraoperative hypotension

**Figure 12:**
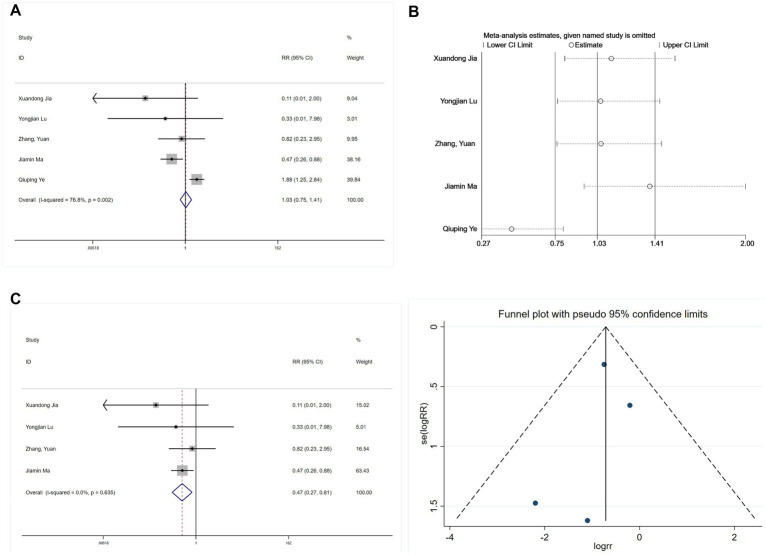
Forest plots and sensitivity analysis for the effect of esketamine on intraoperative hypotension: **(A)** Initial fixed-effects meta-analysis of five studies; **(B)** Sensitivity analysis; **(C)** Final fixed-effects meta-analysis of four studies; and **(D)** Funnel plot assessing publication bias for the final model.

Five studies reported intraoperative hypotension (21, 25, 27, 28, 30). An initial heterogeneity analysis revealed significant variation (I^2^ = 76.8%, *p* = 0.002; Figure 12A). A sensitivity analysis (Figure 12B) identified the study by Ye (28) as a substantial source of heterogeneity, primarily due to its unique use of an opioid-free regimen compared to the opioid-based regimens in other studies. After excluding this study, heterogeneity was negligible (I^2^ = 0%, *p* = 0.635). A subsequent meta-analysis using a fixed-effects model exhibited a significant protective effect of esketamine against intraoperative hypotension (RR = 0.47, 95% CI: 0.27–0.81, *p* = 0.006; Figure 12C). Begg’s test (*p* = 0.734) and funnel plot symmetry (Figure 12D) suggested no publication bias.

###### 3.3.2.3.2 Intraoperative hypertension

**Figure 13:**
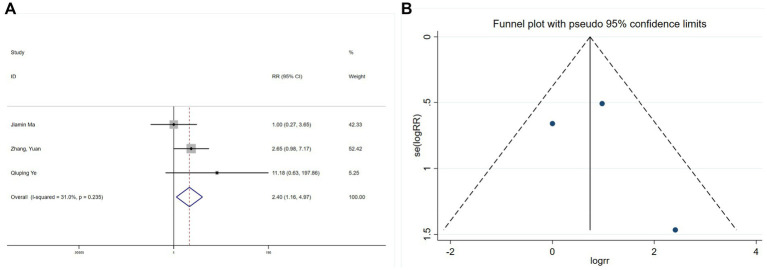
Forest plot and funnel plot for the effect of esketamine on hypertension: **(A)** Fixed-effects meta-analysis of three studies and **(B)** funnel plot assessing publication bias.

Three studies (26, 28, 30) assessed intraoperative hypertension, revealing limited heterogeneity (I^2^ = 31%, *p* = 0.235). A meta-analysis using a fixed-effects model indicated that esketamine significantly increased hypertension incidence (RR = 2.40, 95% CI: 1.16–4.97, *p* = 0.019; Figure 13A). Begg’s test (*p* = 0.1) and funnel plot analysis (Figure 13B) did not suggest publication bias. Although these findings suggest an increased risk of intraoperative hypertension with esketamine, moderate heterogeneity warrants cautious interpretation.

### 3.4 Sensitivity analysis

For outcomes exhibiting low heterogeneity (I^2^ ≤ 50%) or involving ≤ 2 studies, analyses were conducted using both fixed-effects and random-effects models. The comparison (Table 3) revealed consistent pooled effect-size estimates, indicating robust results and minimal influence of the model choice on conclusions.

**Table 3:** Sensitivity analysis.

Outcomes		Fixed-effects model		Random-effects model	
		RR	95% CI	RR	95% CI
POD		0.58	(0.43，0.79)	0.60	(0.44,0.81)
Nausea and vomiting		0.57	(0.44，0.74)	0.60	(0.43，0.84)
Dizziness		1.29	(1.03，1.62)	1.29	(1.01，1.64)
Hallucinations		1.67	(0.62，4.53)	1.66	(0.61，4.53)
Nightmares		1.53	(0.53，4.42)	1.50	(0.49，4.61)
Hypotension		0.47	(0.27，0.81)	0.49	(0.29，0.84)
Hypertension		2.40	(1.16，4.97)	2.13	(0.8,5.64)
MMSE		SMD	95% CI	SMD	95% CI
		0.06	(−0.11,0.24)	0.06	(0.11,0.24)
Remifentanil	Without induction	0.17	(−0.05, 0.38)	0.19	(−0.13,0.51)
	At induction	−0.43	(−0.59,-0.26)	−0.43	(−0.59, −0.26)

**Figure 14:**
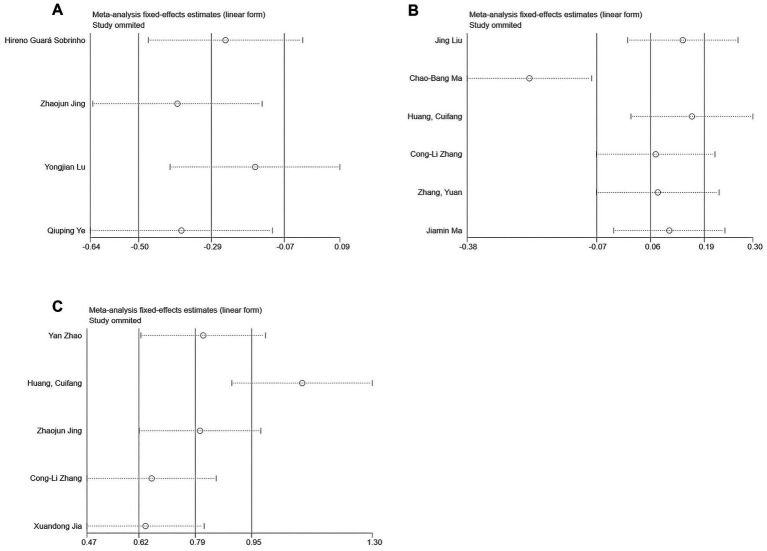
Sensitivity analysis results: **(A)** VAS; **(B)** NRS; and **(C)** QoR-15.

For outcomes with high heterogeneity (I^2^ > 50%) or involving more than two studies, a sensitivity analysis was conducted using the leave-one-out method. As shown in Figure 14, no substantial changes in heterogeneity were observed for NRS or QoR-15 scores. However, significant heterogeneity changes were noted for VAS after excluding studies by Lu et al. (25) or Jing et al. (22). Possible explanations include variations in perioperative opioid use and surgical procedures, although subgroup analyses did not clearly identify a single cause.

### 3.5 Grade assessment

**Figure 15:**
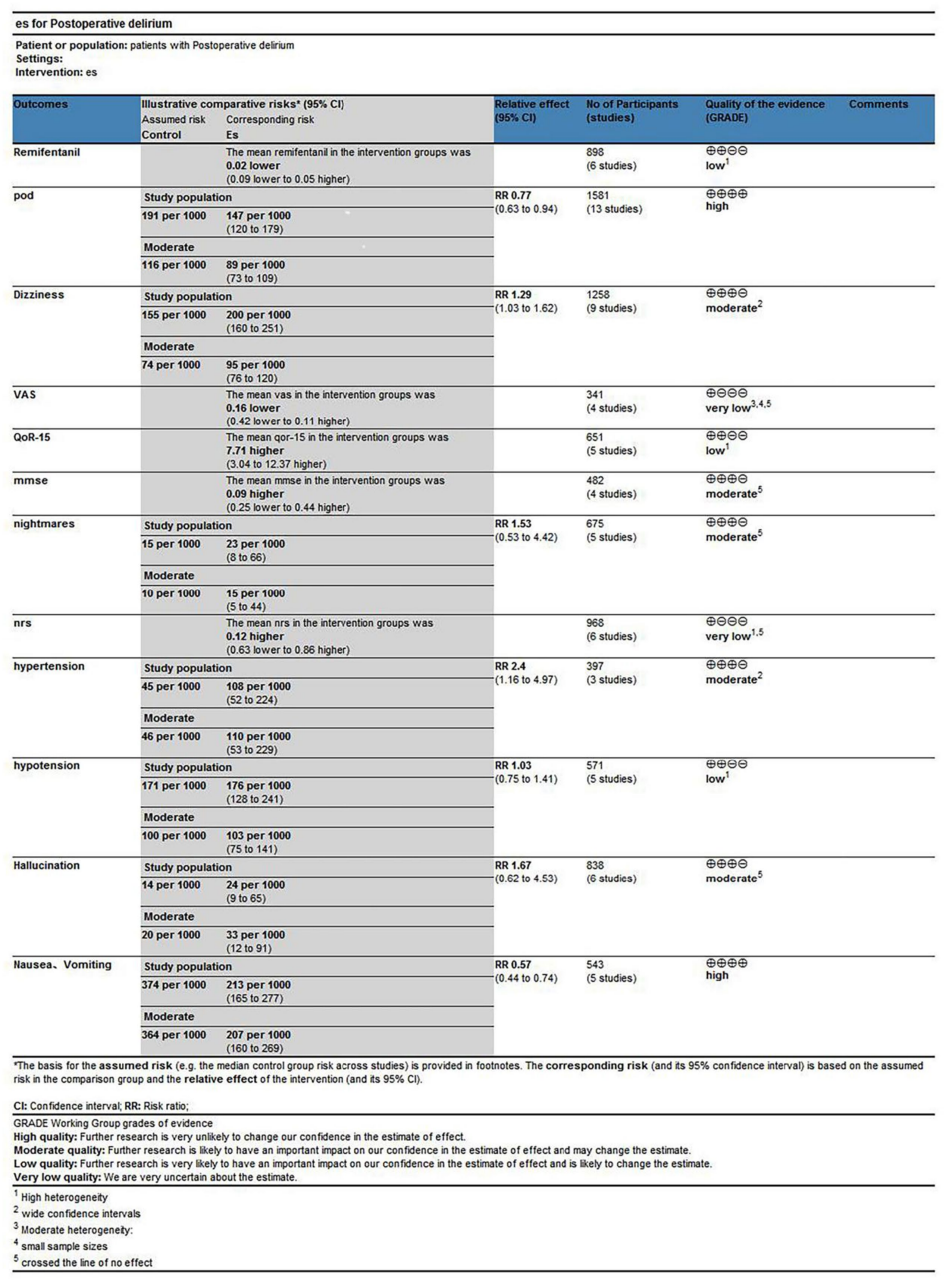
Summary of findings table.

Esketamine administration significantly reduced POD incidence, supported by high-quality evidence. Similarly, reductions in postoperative nausea and vomiting were supported by high-quality evidence. In contrast, evidence indicating increased dizziness incidence was rated moderate quality due to imprecision. Pain-related outcomes (VAS, NRS, and QoR-15) were rated very low to low quality, primarily due to substantial heterogeneity, imprecision, or both, limiting definitive conclusions. For hemodynamic outcomes, evidence suggesting an increased hypertension risk was rated moderate quality, whereas evidence for hypotension was low quality, primarily due to initial heterogeneity, which was mitigated following the sensitivity analysis. Evidence on remifentanil consumption was rated low quality, mainly reflecting high heterogeneity, which improved after the subgroup analysis. Other adverse events, including hallucinations, nightmares, and MMSE outcomes, were supported by moderate-quality evidence but featured wide CI. The Grading of Recommendations, Assessment, Development and Evaluation (GRADE) assessment indicates that although certain benefits are robust, uncertainty remains regarding several outcomes, warranting cautious interpretation (Figure 15).

## 4 Discussion

This meta-analysis, encompassing 13 RCTs involving 1,581 elderly patients, suggests a potential association between perioperative esketamine administration and reduced POD incidence. Notably, after excluding one outlying study (Y. (30)), which identified surgical timing as a significant confounder, heterogeneity disappeared (I^2^ = 0%), indicating remarkable consistency among the remaining studies. According to Zhang et al., heterogeneity primarily increased from differences in surgical timing; afternoon surgeries independently reduced POD risk, irrespective of esketamine use. The other studies did not specifically control for surgical timing. Although variability in POD diagnostic tools might contribute to heterogeneity, 11 of the 13 included trials used the Confusion Assessment Method (CAM or CAM-ICU), the accepted criterion standard. Two studies (19, 21) did not specify their diagnostic instruments. Sensitivity analysis excluding studies with unspecified tools reinforced the robustness of the primary association. Future perioperative studies should consistently use and explicitly report standardized diagnostic tools such as CAM to improve comparability. Han et al. (32) reviewed previous studies examining esketamine’s effect on postoperative cognitive dysfunction in elderly patients, concluding that esketamine delayed neurocognitive recovery (dNCR) at 30 days postoperatively, despite improvements in early cognitive function. Another meta-analysis (33) indicated no clear preventive effect of perioperative esketamine on POD or postoperative neurocognitive disorder (POND), but considerable heterogeneity was present. Cognitive function differences across elderly surgical patients (aged 60–80 years) may partly explain this inconsistency. Although sensitivity analysis excluding age-related studies did not fundamentally alter conclusions, detailed subgroup analyses based on age should be pursued to enhance clinical applicability. The current literature regarding esketamine and neurocognitive outcomes remains inconclusive, with some reviews indicating no preventive effects or even potential risks. Our analysis, while consistently showing a beneficial direction, contributes to this evolving and uncertain evidence base. Therefore, further investigation into esketamine’s effect on POD in high-risk populations using standardized methodologies is necessary. Future studies are required to confirm potential benefits and determine optimal dosing and timing strategies.

In this study, esketamine primarily reduced the incidence of early POD in elderly patients, supported by relevant mechanistic evidence. Earlier research has revealed that esketamine decreases plasma levels of S100 calcium-binding protein β (S100β), a widely recognized biomarker for brain injury (34). Numerous perioperative factors, including surgical stress, anesthetic effects, blood–brain barrier dysfunction, and epigenetic modifications, can induce neuroinflammation, a critical mechanism underlying POD pathogenesis (35). Esketamine has also been shown to suppress neuroinflammation and neuronal apoptosis by reducing S100β expression (6). Animal studies have indicated that esketamine significantly attenuates neuroinflammation, thereby potentially preventing delirium (36). Thus, the association suggested by our meta-analysis is biologically plausible, as mechanistic studies indicate that esketamine may reduce neuroinflammation and brain injury markers, both implicated in POD pathogenesis. These findings offer a supportive rationale for the clinical hypothesis generated by our statistical analyses. Furthermore, this study provides clinically practical absolute effect measures. An NNT of 16 (95% CI: 16–29) indicates moderate clinical utility of esketamine for POD prevention. Importantly, this NNT is calculated based on an overall baseline POD risk of 13%. In high-risk populations (baseline risk ≥30%), the NNT would decrease to approximately 10, enhancing the value of this intervention, whereas its utility diminishes among lower-risk patients. This supports the rationale for targeted interventions in high-risk individuals.

Contrary to previous reports, this meta-analysis did not find a significant analgesic benefit from the administration of esketamine. Previous research often showed esketamine’s effectiveness in providing short-term post-surgical analgesia (37, 38). Esketamine’s analgesic effect primarily relies on its NMDA receptor antagonism and prevention of nociceptive signaling at the dorsal horn of the spinal cord. In addition, esketamine can deliver analgesic effects through peripheral NMDA receptor modulation (39). The inconsistent analgesic findings in the current analysis could be due to varied pain perception thresholds in geriatric populations. In one previous study (6), no significant differences in postoperative pain scores were reported between older adults and younger adults when assessed at rest and during mobilization in the first 48 post-surgical h.

Our findings must be interpreted within the broader context of multi-component delirium prevention strategies. Dexmedetomidine, a widely recognized pharmacologic agent, effectively reduces POD incidence but has not consistently demonstrated superiority regarding critical outcomes such as in-hospital mortality, long-term mortality, delirium duration, or mechanical ventilation duration (40, 41). Within this context, esketamine’s opioid-sparing effect potentially mitigates opioid-associated adverse effects such as respiratory depression, which may contribute to delirium. However, these potential benefits must be balanced against esketamine’s adverse effect profile, notably increased risks of hypertension and dizziness, limiting its application among vulnerable populations. Lee et al. (42) have also indicated that combination therapies appear more effective in preventing POD, underscoring the importance of determining optimal pharmacological approaches. Importantly, multidisciplinary strategies integrating both pharmacological and non-pharmacological interventions have shown superior efficacy in preventing and managing POD among older adults (43). Thus, esketamine should be considered not as a standalone intervention but as a potential pharmacological adjunct within comprehensive delirium prevention strategies.

An interesting finding of this research was that esketamine administration at induction affected remifentanil usage. Studies conducted by Yuan et al. (44) have shown that continuous administration at the rate of 0.250 mg·kg^−1^·h^−1^ decreased opioid consumption. Stress responses in geriatric patients are alleviated by esketamine, serving as a primary mechanism for reducing opioid use (38). Due to differences in surgical stress across operative phases, administering esketamine at induction may facilitate more comprehensive control of perioperative stress and optimize opioid usage. However, the lack of clear clinical recommendations, such as specific dosing ranges or clearly defined indications and contraindications, limits its direct clinical application. Therefore, future studies should focus on establishing evidence-based protocols for optimal dosing, timing, and patient selection in perioperative esketamine administration.

Esketamine administration did not significantly improve MMSE scores, nor did it reduce postoperative hallucinations, dizziness, or perioperative nightmares, aligning with the majority of previous studies (45, 46). Dizziness in elderly patients may pose significant risks, particularly by increasing the likelihood of falls, fractures, and delayed postoperative mobilization, thereby adversely affecting overall recovery (47). Nevertheless, owing to its unique pharmacology compared to classical ketamine, esketamine significantly reduced postoperative nausea and vomiting (8, 48). This finding, considering the susceptibility of the older population, stands out among the main points in need of clinical improvement.

QoR-15 scores suggested that esketamine could enhance postoperative sleep quality, consistent with the majority of peer-reviewed studies (49, 50). However, significant heterogeneity in this meta-analysis must be interpreted with caution. Future high-standard studies are needed to confirm these results, thereby enhancing the evidentiary base for clinical practice decisions.

Esketamine showed benefits in the control of intraoperative hypotension but increased the risk of hypertension. Intraoperative hypotension analysis revealed heterogeneity due to an unusual study design adopted by the Qiuping Ye (28) team. Their experimental group used an opioid-free regimen with esketamine alone, which deviated from other studies. Compared to ketamine, esketamine more effectively sustains perioperative hemodynamic stability (51), which can be explained by its mechanism of action (52). Esketamine likely blocks sodium channels at brainstem parasympathetic neurons, inhibits parasympathetic cardiac nerve activity, raises cardiac output, inhibits neuronal norepinephrine uptake, and increases systemic norepinephrine levels. Such mechanisms elevate sympathetic excitability and peripheral vascular resistance, resulting in stable hemodynamics. Compared to the control group, patients receiving esketamine experienced a 2.40-fold increased risk of intraoperative hypertension. For elderly surgical patients, who frequently have underlying hypertension, atherosclerosis, or cardiovascular comorbidities (e.g., coronary heart disease, heart failure, or stroke history), acute intraoperative hypertension is a well-established risk factor for severe perioperative complications, including myocardial ischemia, arrhythmias, acute heart failure, stroke, and intracranial hemorrhage (53). Therefore, when administering esketamine, clinicians should carefully balance its analgesic and anti-delirium benefits against the associated risks of hemodynamic instability. We recommend rigorous real-time blood pressure monitoring and readiness for immediate antihypertensive intervention in these patients.

Several limitations must be acknowledged when interpreting these results. First, substantial variability was observed across studies concerning esketamine dosing (ranging from 0.2 to 1.0 mg/kg), timing of administration (e.g., induction vs. postoperative analgesia), administration methods, and concomitant analgesic use. Such variability, although reflective of ongoing clinical exploration, inevitably reduces the comparability of pooled data and complicates definitive conclusions regarding optimal perioperative protocols. Second, in this current study, the incidence of POD was evaluated only on the first postoperative day. Future studies should include multiple postoperative time points for comparison. Third, although the clinical advantages of esketamine are evident, studies are required to determine optimal dosing regimens. Variability in physiological state, degree of stress, and therapeutic needs across perioperative periods (induction, maintenance, and postoperative analgesia) can influence esketamine efficacy, dosing, and safety. Finally, the majority of studies included were from China, which may limit generalizability due to ethnic differences. However, we suggest that while our findings remain robust within the studied context, this meta-analysis provides foundational insights into the clinical use of esketamine. Therefore, these results should be interpreted as indicative of general trends and safety profiles of esketamine across various clinical practices rather than as evidence supporting a specific protocol.

## 5 Conclusion

This meta-analysis indicates a potential net benefit of esketamine in perioperative care for elderly patients, demonstrating improvements in several key recovery domains. However, due to notable heterogeneity in some analyses (e.g., pain scores and QoR-15), the certainty of evidence is moderate, and findings should be interpreted cautiously. Esketamine administration was associated with reductions in POD, nausea, and vomiting. Additionally, it showed potential in stabilizing perioperative hemodynamics by mitigating hypotension, although it may concurrently increase the risk of hypertension and dizziness. Benefits in postoperative sleep quality were also observed. Moreover, esketamine administered at induction reduced intraoperative opioid consumption, suggesting a role in optimizing perioperative analgesia. Given its multifaceted and variable effect profile, the overall risk–benefit ratio of esketamine should be evaluated individually, supporting personalized strategies for optimizing perioperative analgesia.

Future studies should concentrate on identifying optimal timing and regimens for esketamine administration to maximize therapeutic benefits and minimize risks, particularly hemodynamic instability. Extended follow-up is also essential to assess long-term impacts on cognitive trajectories, functional independence, and quality of life. Such evidence is critical for guiding precise and individualized perioperative management strategies in elderly surgical patients.

## Data Availability

The datasets presented in this study can be found in online repositories. The names of the repository/repositories and accession number(s) can be found in the article/Supplementary material.
