# Supplementary material for: A comprehensive review and meta-analysis on the safety and efficacy of esketamine for emerging delirium in elderly patients
Source: Front Med (Lausanne). 2026 Feb 6;13:1752055. doi: 10.3389/fmed.2026.1752055 (PMC12921487; doi:10.3389/fmed.2026.1752055)

Table 1 search strategy of PubMed

| Search number | Query | Results |
| --- | --- | --- |
| #1 | "Emergence Delirium"[Mesh] | 1005 |
| #2 | (((((((((((((((((Delirium, Emergence[Title/Abstract]) OR (Agitated Emergence[Title/Abstract])) OR (Emergence, Agitated[Title/Abstract])) OR (Emergence Agitation[Title/Abstract])) OR (Agitation, Emergence[Title/Abstract])) OR (Agitations, Emergence[Title/Abstract])) OR (Emergence Excitement[Title/Abstract])) OR (Excitement, Emergence[Title/Abstract])) OR (Postanesthetic Excitement[Title/Abstract])) OR (Excitement, Postanesthetic[Title/Abstract])) OR (Anesthesia Emergence Delirium[Title/Abstract])) OR (Delirium, Anesthesia Emergence[Title/Abstract])) OR (Emergence Delirium, Anesthesia[Title/Abstract])) OR (Delirium, Postoperative[Title/Abstract])) OR (Post-Operative Delirium[Title/Abstract])) OR (Delirium, Post-Operative[Title/Abstract])) OR (Post Operative Delirium[Title/Abstract])) OR (Postoperative Delirium[Title/Abstract]) | 5386 |
| #3 | "Esketamine" [Supplementary Concept] | 827 |
| #4 | ((((L-Ketamine[Title/Abstract]) OR ((-)-Ketamine[Title/Abstract])) OR (S-Ketamine[Title/Abstract])) OR ((S)-2-(o-chlorophenyl)-2-(methylamino)cyclohexanone[Title/Abstract])) OR (Spravato[Title/Abstract]) | 24991 |
| #5 | Kataved[Title/Abstract] - Schema: all | 0 |
| #6 | Kataved[Title/Abstract] | 0 |
| #7 | "Ketamine"[Mesh] | 16567 |
| #8 | ((((((((((2-(2-Chlorophenyl)-2-(methylamino)cyclohexanone[Title/Abstract]) OR (Ketalar[Title/Abstract])) OR (CI-581[Title/Abstract])) OR (CI581[Title/Abstract])) OR (CI 581[Title/Abstract])) OR (Ketanest[Title/Abstract])) OR (Ketamine Hydrochloride[Title/Abstract])) OR (Calipsol[Title/Abstract])) OR (Calypsol[Title/Abstract])) OR (Kalipsol[Title/Abstract])) OR (Ketaset[Title/Abstract]) | 1082 |
| #9 | (Aged[Title/Abstract]) OR (Elderly[Title/Abstract]) | 1114410 |
| #10 | ((randomized controlled trial[Publication Type]) OR (placebo[Title/Abstract])) OR (randomized[Title/Abstract]) | 1139835 |
| #11 | ("Emergence Delirium"[Mesh]) OR ((((((((((((((((((Delirium, Emergence[Title/Abstract]) OR (Agitated Emergence[Title/Abstract])) OR (Emergence, Agitated[Title/Abstract])) OR (Emergence Agitation[Title/Abstract])) OR (Agitation, Emergence[Title/Abstract])) OR (Agitations, Emergence[Title/Abstract])) OR (Emergence Excitement[Title/Abstract])) OR (Excitement, Emergence[Title/Abstract])) OR (Postanesthetic Excitement[Title/Abstract])) OR (Excitement, Postanesthetic[Title/Abstract])) OR (Anesthesia Emergence Delirium[Title/Abstract])) OR (Delirium, Anesthesia Emergence[Title/Abstract])) OR (Emergence Delirium, Anesthesia[Title/Abstract])) OR (Delirium, Postoperative[Title/Abstract])) OR (Post-Operative Delirium[Title/Abstract])) OR (Delirium, Post-Operative[Title/Abstract])) OR (Post Operative Delirium[Title/Abstract])) OR (Postoperative Delirium[Title/Abstract])) | 5481 |
| #12 | ((("Esketamine" [Supplementary Concept]) OR (((((L-Ketamine[Title/Abstract]) OR ((-)-Ketamine[Title/Abstract])) OR (S-Ketamine[Title/Abstract])) OR ((S)-2-(o-chlorophenyl)-2-(methylamino)cyclohexanone[Title/Abstract])) OR (Spravato[Title/Abstract]))) OR ("Ketamine"[Mesh])) OR (((((((((((2-(2-Chlorophenyl)-2-(methylamino)cyclohexanone[Title/Abstract]) OR (Ketalar[Title/Abstract])) OR (CI-581[Title/Abstract])) OR (CI581[Title/Abstract])) OR (CI 581[Title/Abstract])) OR (Ketanest[Title/Abstract])) OR (Ketamine Hydrochloride[Title/Abstract])) OR (Calipsol[Title/Abstract])) OR (Calypsol[Title/Abstract])) OR (Kalipsol[Title/Abstract])) OR (Ketaset[Title/Abstract])) | 27300 |
| #13 | ((((Aged[Title/Abstract]) OR (Elderly[Title/Abstract])) AND (((randomized controlled trial[Publication Type]) OR (placebo[Title/Abstract])) OR (randomized[Title/Abstract]))) AND (("Emergence Delirium"[Mesh]) OR ((((((((((((((((((Delirium, Emergence[Title/Abstract]) OR (Agitated Emergence[Title/Abstract])) OR (Emergence, Agitated[Title/Abstract])) OR (Emergence Agitation[Title/Abstract])) OR (Agitation, Emergence[Title/Abstract])) OR (Agitations, Emergence[Title/Abstract])) OR (Emergence Excitement[Title/Abstract])) OR (Excitement, Emergence[Title/Abstract])) OR (Postanesthetic Excitement[Title/Abstract])) OR (Excitement, Postanesthetic[Title/Abstract])) OR (Anesthesia Emergence Delirium[Title/Abstract])) OR (Delirium, Anesthesia Emergence[Title/Abstract])) OR (Emergence Delirium, Anesthesia[Title/Abstract])) OR (Delirium, Postoperative[Title/Abstract])) OR (Post-Operative Delirium[Title/Abstract])) OR (Delirium, Post-Operative[Title/Abstract])) OR (Post Operative Delirium[Title/Abstract])) OR (Postoperative Delirium[Title/Abstract])))) AND (((("Esketamine" [Supplementary Concept]) OR (((((L-Ketamine[Title/Abstract]) OR ((-)-Ketamine[Title/Abstract])) OR (S-Ketamine[Title/Abstract])) OR ((S)-2-(o-chlorophenyl)-2-(methylamino)cyclohexanone[Title/Abstract])) OR (Spravato[Title/Abstract]))) OR ("Ketamine"[Mesh])) OR (((((((((((2-(2-Chlorophenyl)-2-(methylamino)cyclohexanone[Title/Abstract]) OR (Ketalar[Title/Abstract])) OR (CI-581[Title/Abstract])) OR (CI581[Title/Abstract])) OR (CI 581[Title/Abstract])) OR (Ketanest[Title/Abstract])) OR (Ketamine Hydrochloride[Title/Abstract])) OR (Calipsol[Title/Abstract])) OR (Calypsol[Title/Abstract])) OR (Kalipsol[Title/Abstract])) OR (Ketaset[Title/Abstract]))) | 55 |

Table 2 search strategy of EMBASE

| Search number | Query | Results |
| --- | --- | --- |
| #1 | 'emergence agitation'/exp | 1,807 |
| #2 | 'emergence delirium':ab,ti OR 'delirium, emergence':ab,ti OR 'agitated emergence':ab,ti OR 'emergence, agitated':ab,ti OR 'agitation, emergence':ab,ti OR 'agitations, emergence':ab,ti OR 'emergence excitement':ab,ti OR 'itement, emergence':ab,ti OR 'postanesthetic excitement':ab,ti OR 'excitement, postanesthetic':ab,ti OR 'anesthesia emergence delirium':ab,ti OR 'delirium, anesthesia emergence':ab,ti OR 'emergence delirium, anesthesia':ab,ti OR 'postoperative delirium':ab,ti OR 'delirium, postoperative':ab,ti OR 'post-operative delirium':ab,ti OR 'delirium, post-operative':ab,ti OR 'post operative delirium':ab,ti | 6,853 |
| #3 | 'esketamine'/exp | 3,226 |
| #4 | 'l ketamine':ab,ti OR (-:ab,ti AND -ketamine:ab,ti) OR 's ketamine':ab,ti | 1,470 |
| #5 | s:ab,ti AND 'o chlorophenyl':ab,ti AND -2-:ab,ti AND methylamino:ab,ti AND cyclohexanone:ab,ti OR kataved:ab,ti OR spravato:ab,ti | 67 |
| #6 | #1 OR #2 | 7,658 |
| #7 | #3 OR #4 OR #5 | 4,199 |
| #8 | 'ketamine'/exp | 76,141 |
| #9 | 2-:ab,ti AND '2 chlorophenyl':ab,ti AND -2-:ab,ti AND methylamino:ab,ti AND cyclohexanone:ab,ti OR ketalar:ab,ti OR ci581:ab,ti OR 'ci 581':ab,ti OR ketanest:ab,ti OR 'ketamine hydrochloride':ab,ti OR calipsol:ab,ti OR calypsol:ab,ti OR kalipsol:ab,ti OR ketaset:ab,ti | 1,380 |
| #10 | 'aged'/exp OR 'aged' | 6,799,210 |
| #11 | elderly:ab,ti | 460,944 |
| #12 | #10 OR #11 | 6,880,259 |
| #13 | 'randomized controlled trial':it OR randomized:ab,ti OR placebo:ab,ti | 1,495,611 |
| #14 | #7 OR #8 OR #9 | 78,322 |
| #15 | #6 AND #12 AND #13 AND #14 | 103 |

Table 3 search strategy of WOS

| Search number | Query | Results |
| --- | --- | --- |
| #1 | ((((((((((((((((((((TS=(Emergence Delirium)) OR TS=(Delirium, Emergence)) OR TS=(Delirium, Emergence)) OR TS=(Agitated Emergence)) OR TS=(Emergence, Agitated)) OR TS=(Emergence Delirium)) OR TS=(Emergence Agitation)) OR TS=(Agitation, Emergence)) OR TS=(Agitations, Emergence)) OR TS=(Emergence Excitement)) OR TS=(Excitement, Emergence)) OR TS=(Postanesthetic Excitement)) OR TS=(Excitement, Postanesthetic)) OR TS=(Anesthesia Emergence Delirium)) OR TS=(Delirium, Anesthesia Emergence)) OR TS=(Emergence Delirium, Anesthesia)) OR TS=(Delirium, Postoperative)) OR TS=(Post-Operative Delirium)) OR TS=(Delirium, Post-Operative)) OR TS=(Post Operative Delirium)) OR TS=(Postoperative Delirium) and Preprint Citation Index (Exclude -Database) | 13185 |
| #2 | (((((((((((((((((TS=(Esketamine)) OR TS=(L-Ketamine)) OR TS=(S-Ketamine)) OR TS=((S)-2-(o-chlorophenyl)-2-(methylamino)cyclohexanone)) OR TS=(Kataved)) OR TS=(Spravato)) OR TS=(Ketamine)) OR TS=(2-(2-Chlorophenyl)-2-(methylamino)cyclohexanone)) OR TS=(Ketalar)) OR TS=(CI-581)) OR TS=(CI581)) OR TS=(CI 581)) OR TS=(Ketamine Hydrochloride)) OR TS=(Calipsol)) OR TS=(Calypsol)) OR TS=(Kalipsol)) OR TS=(Ketaset)) OR TS=(Ketanest) and Preprint Citation Index (Exclude- Database) | 48873 |
| #3 | (TS=( Aged)) OR TS=(Elderly) and Preprint Citation Index (Exclude - Database) | 12496204 |
| #4 | ((TS=(randomized controlled trial)) OR TS=(placebo)) OR TS=(randomized) and Preprint Citation Index (Exclude- Database) | 1807906 |
| #5 | #1 AND #2 AND #3 AND #4 and Preprint Citation Index (Exclude- Database) | 156 |

Table 4 search strategy of OVID


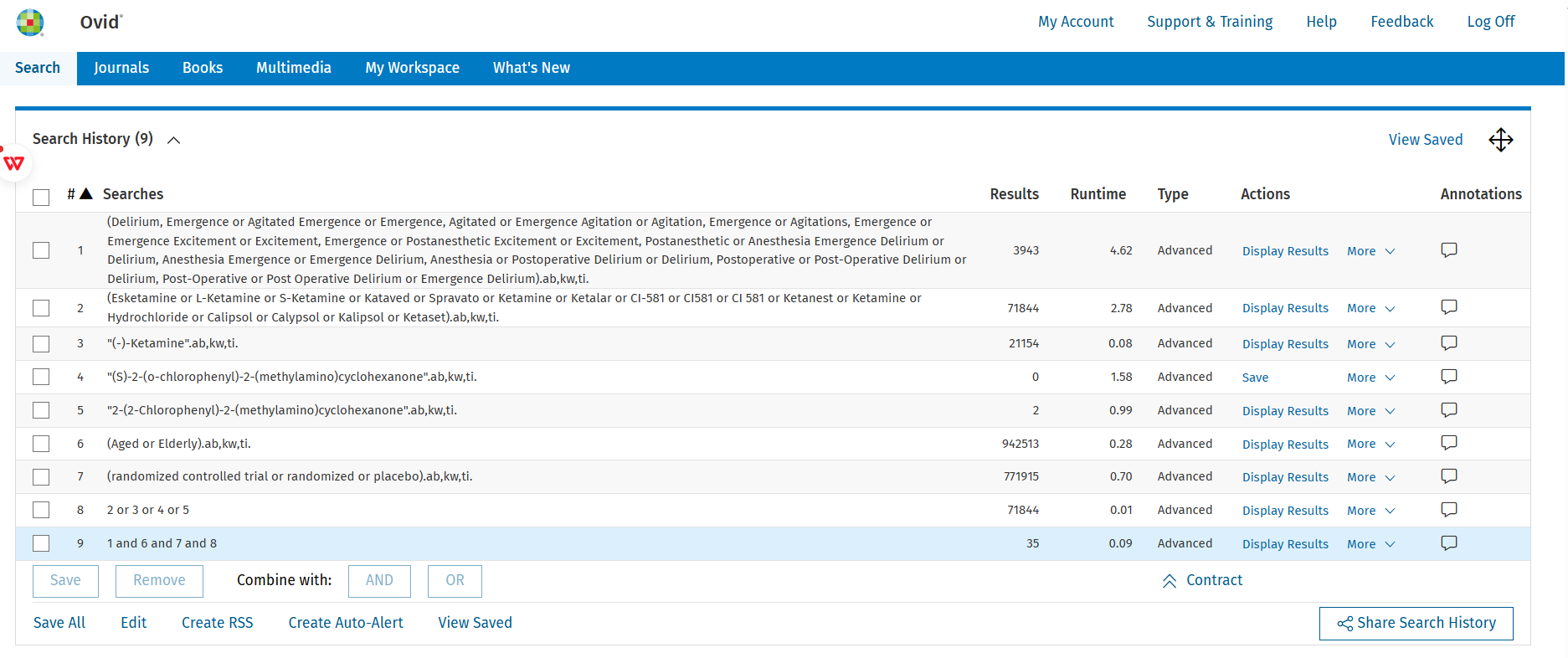


Table 5 search strategy of Cochrane


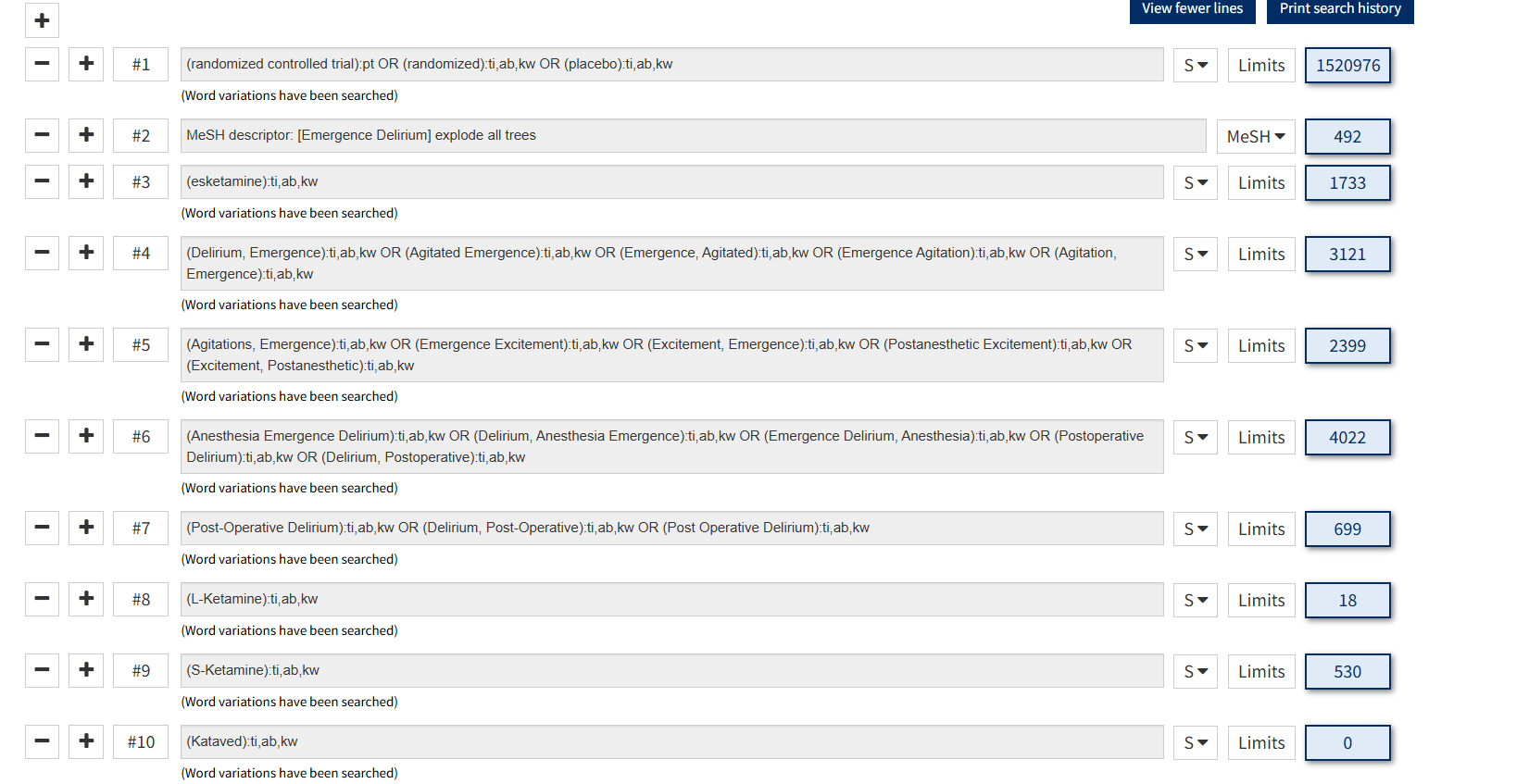

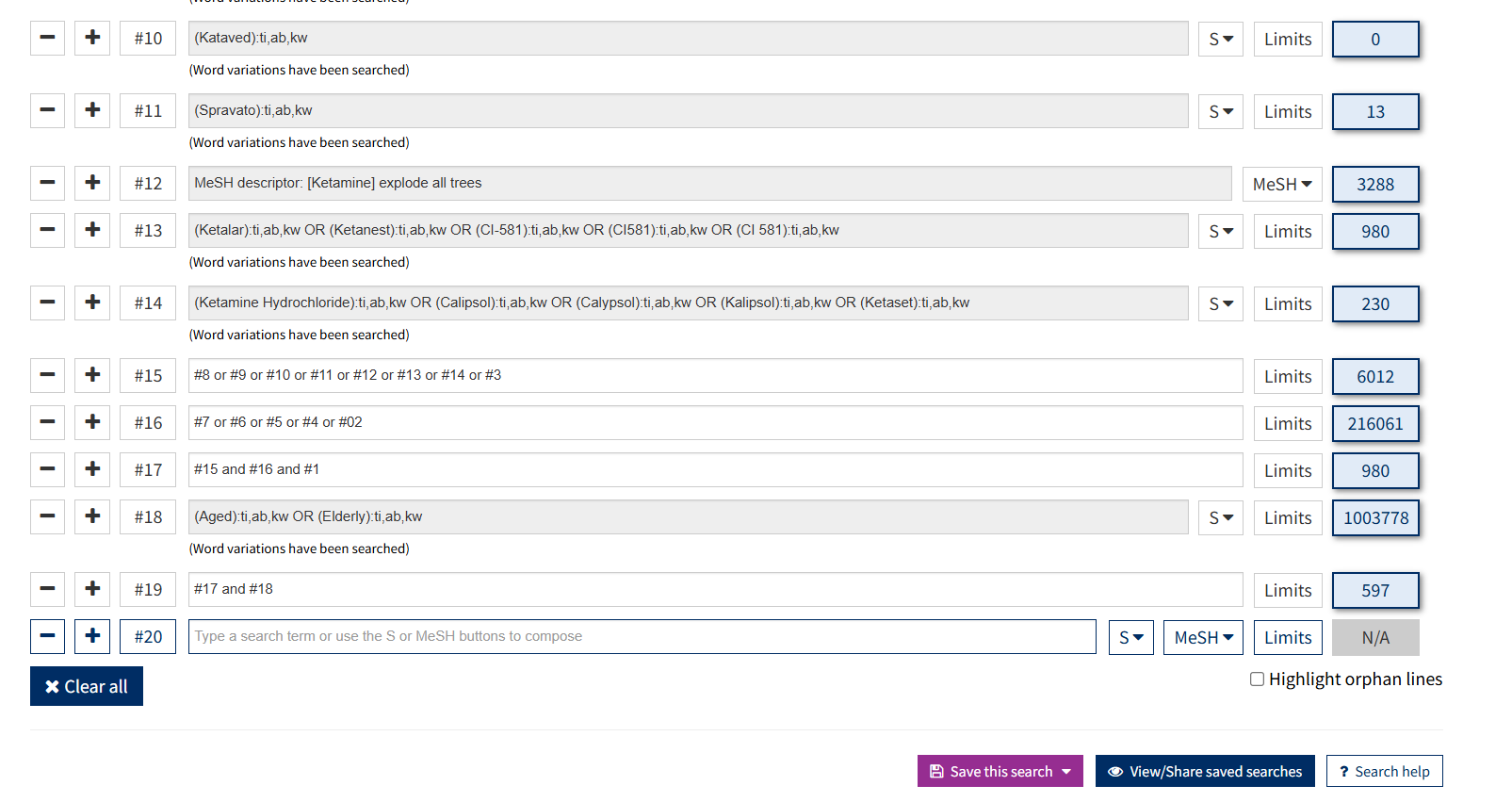


Table 5 search strategy of Scopus
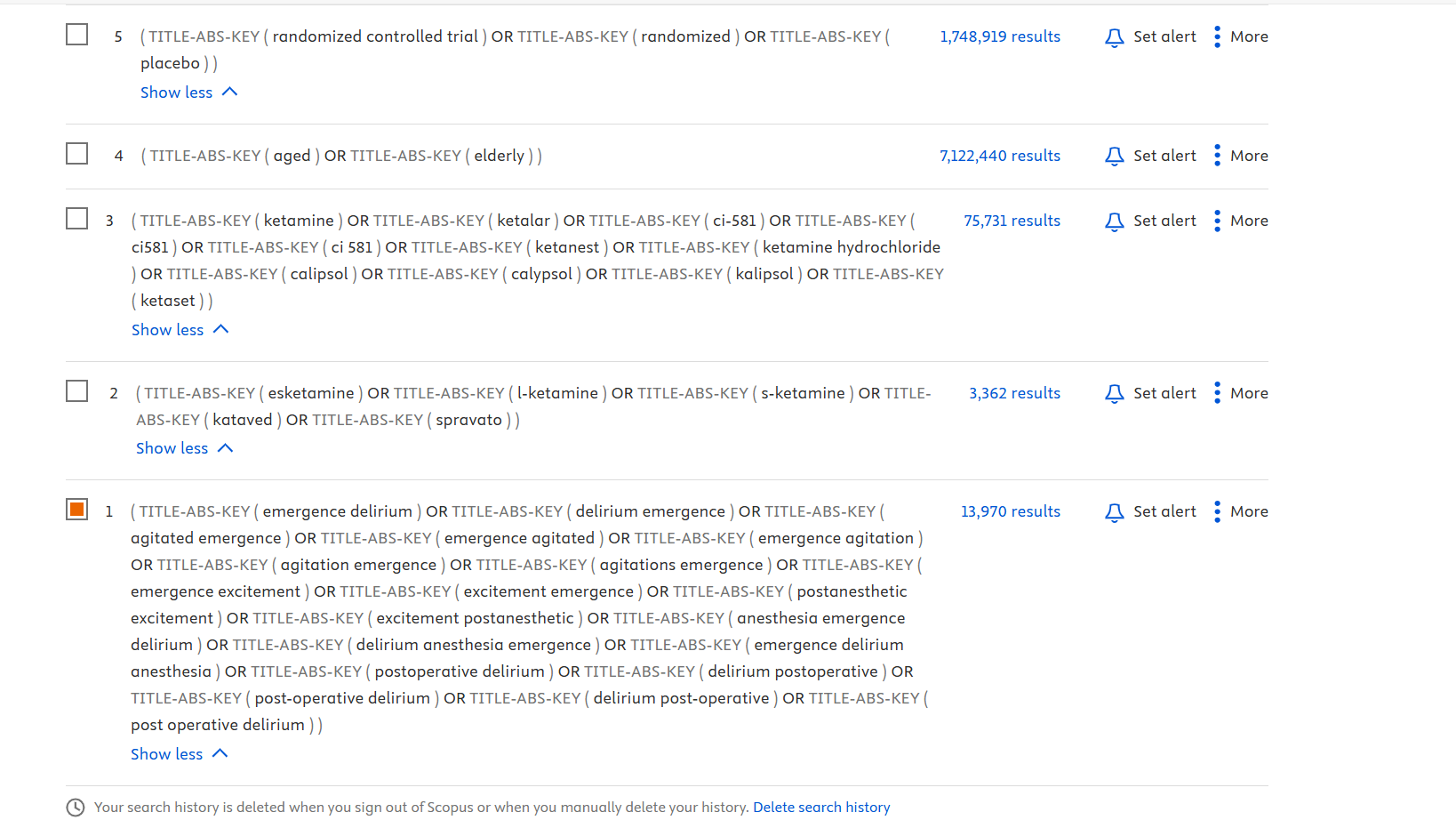


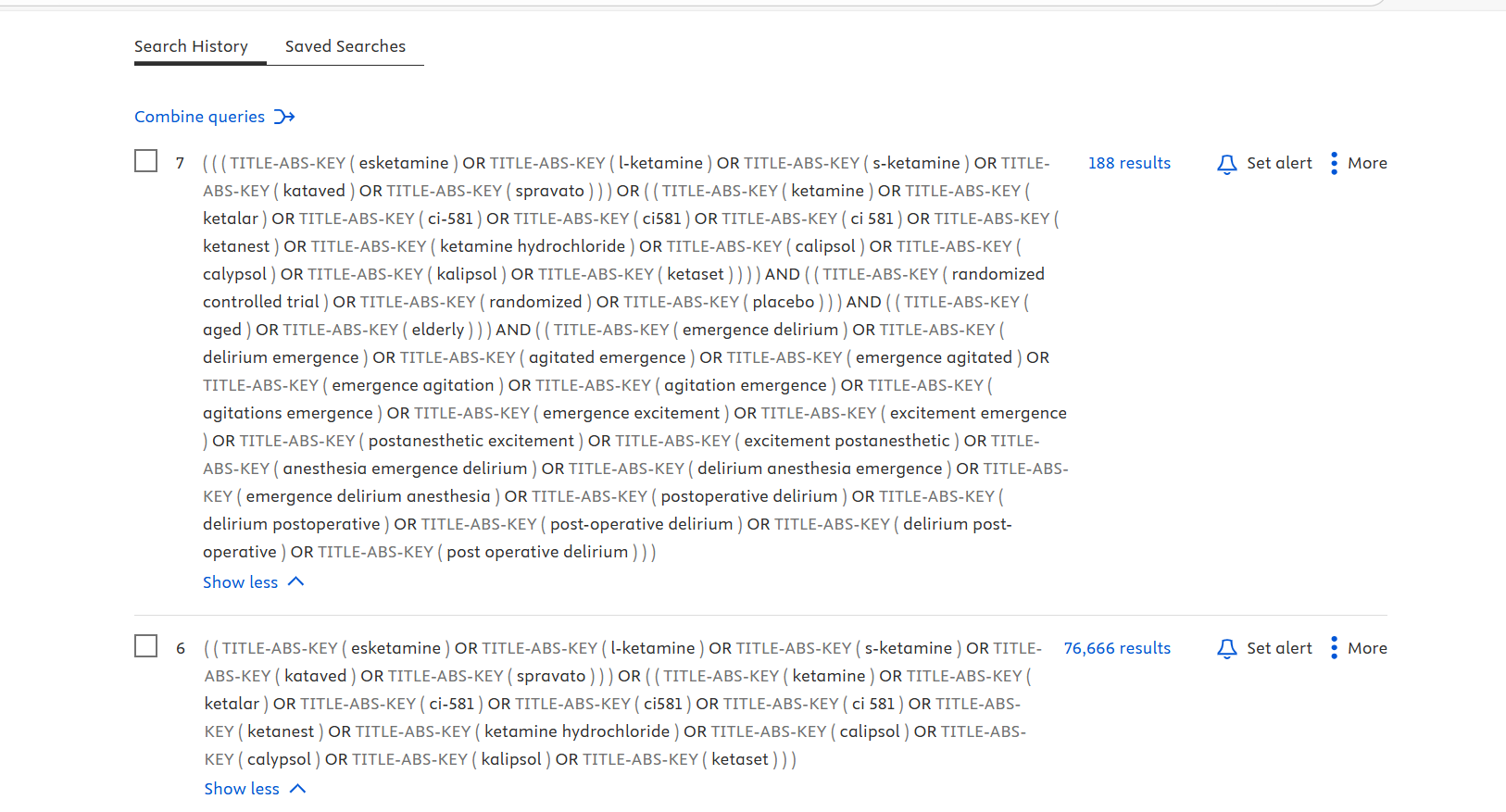

Supplement: Supplementary file 1 [file Table_1.docx]
