# Supplementary figures and images for: A comprehensive review and meta-analysis on the safety and efficacy of esketamine for emerging delirium in elderly patients
Source: Front Med (Lausanne). 2026 Feb 6;13:1752055. doi: 10.3389/fmed.2026.1752055 (PMC12921487; doi:10.3389/fmed.2026.1752055)

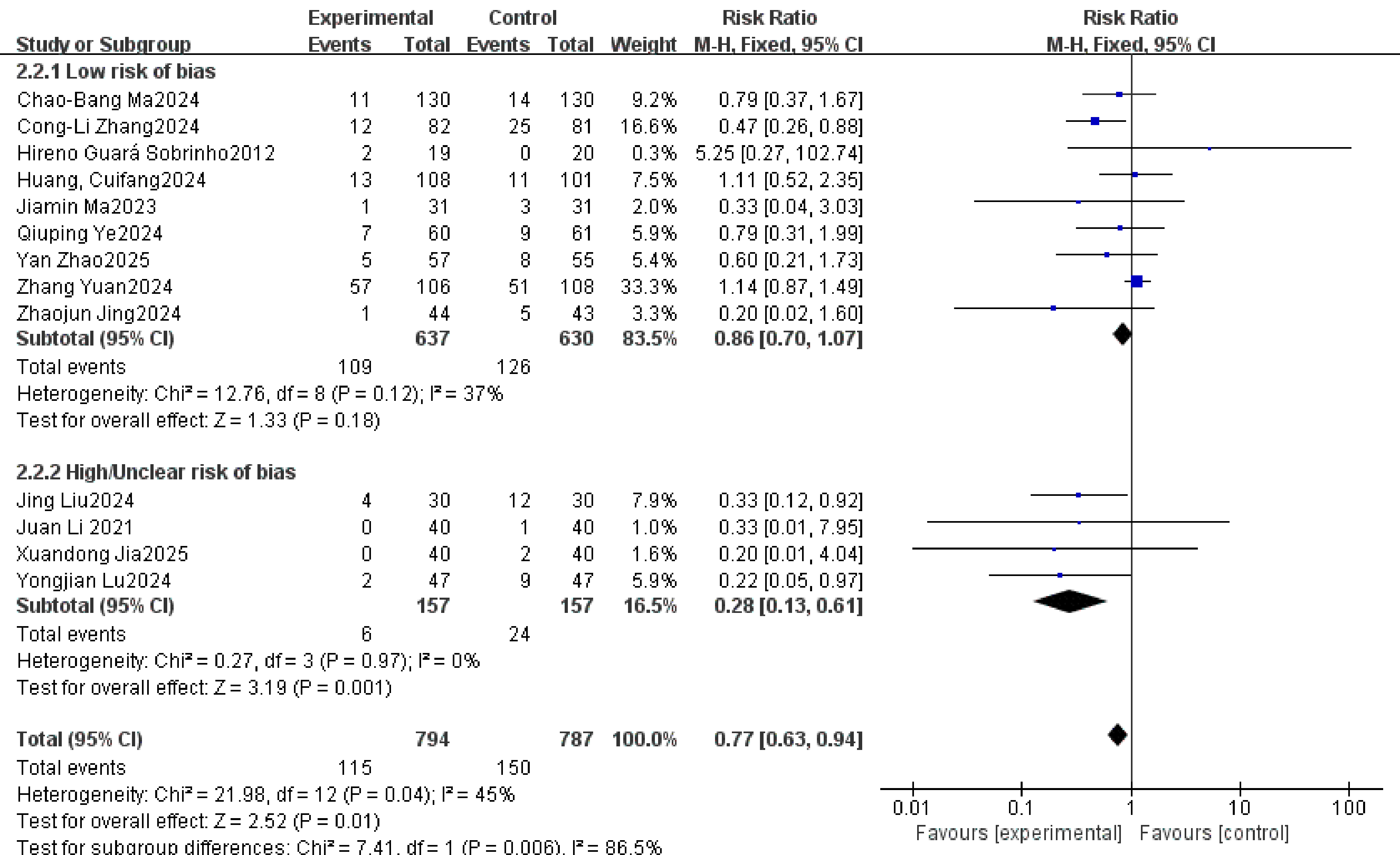

Supplement: Supplementary file 2 [file Image_1.png]

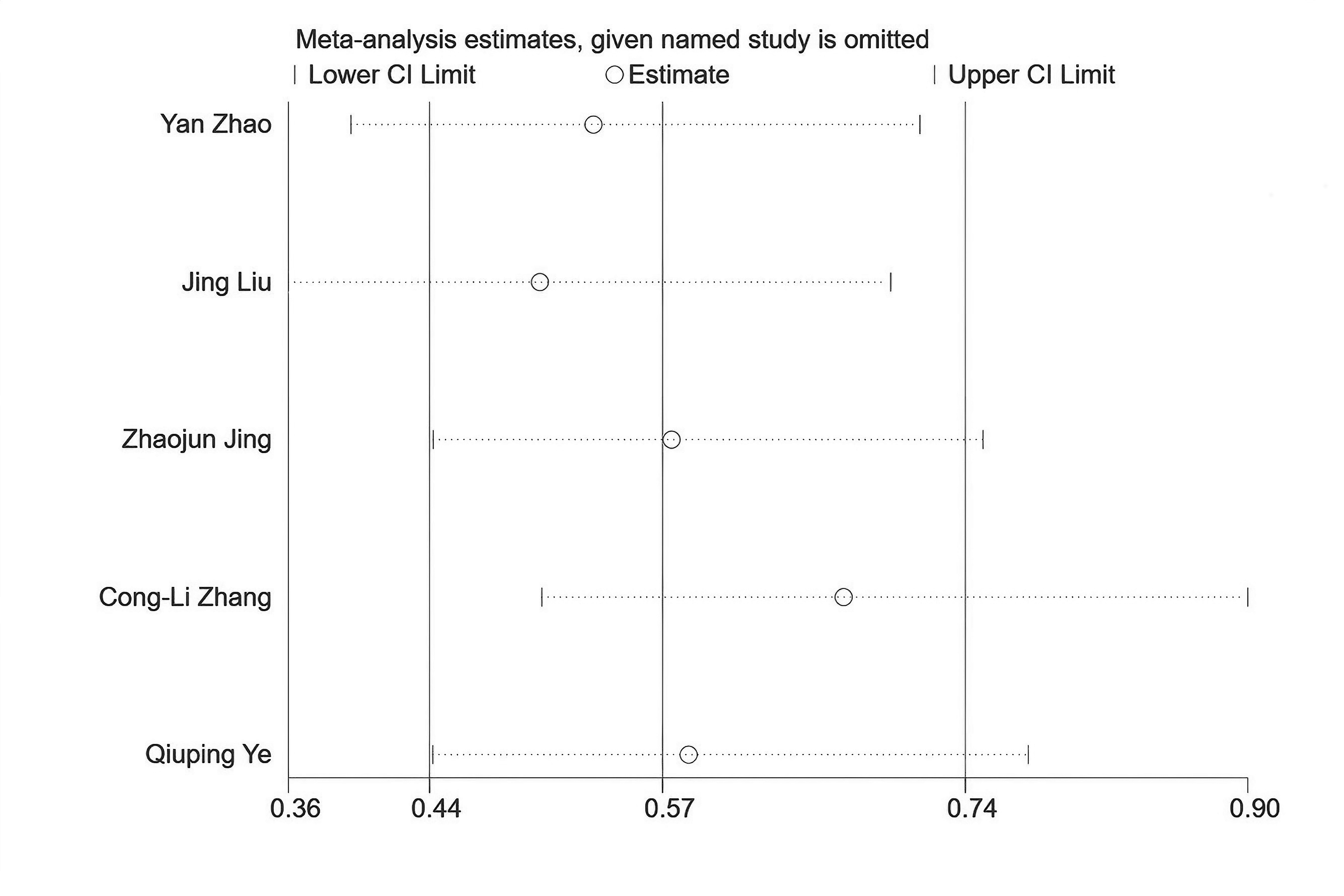

Supplement: Supplementary file 3 [file Image_2.jpeg]

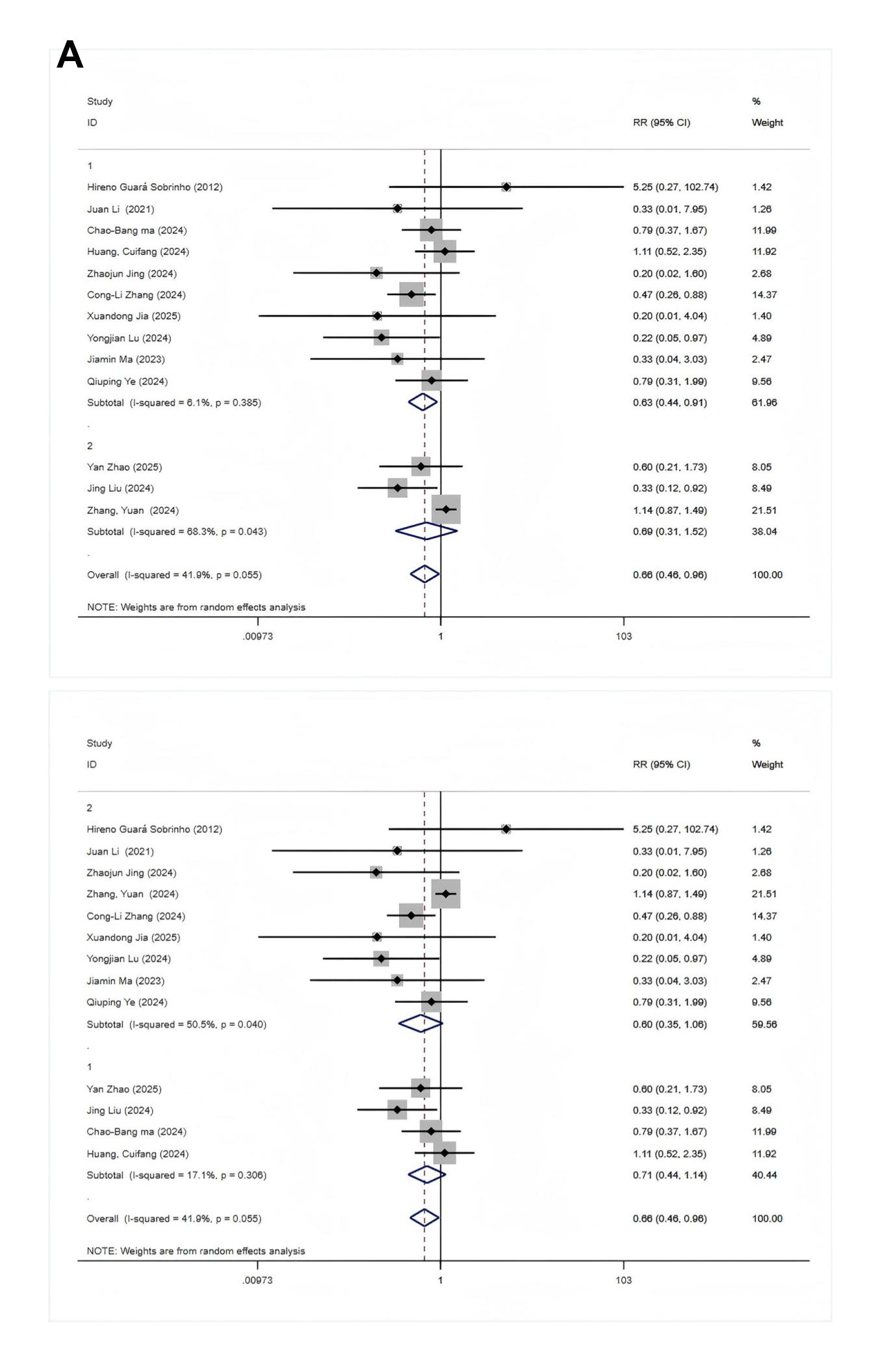

Supplement: Supplementary file 4 [file Image_3.jpeg]

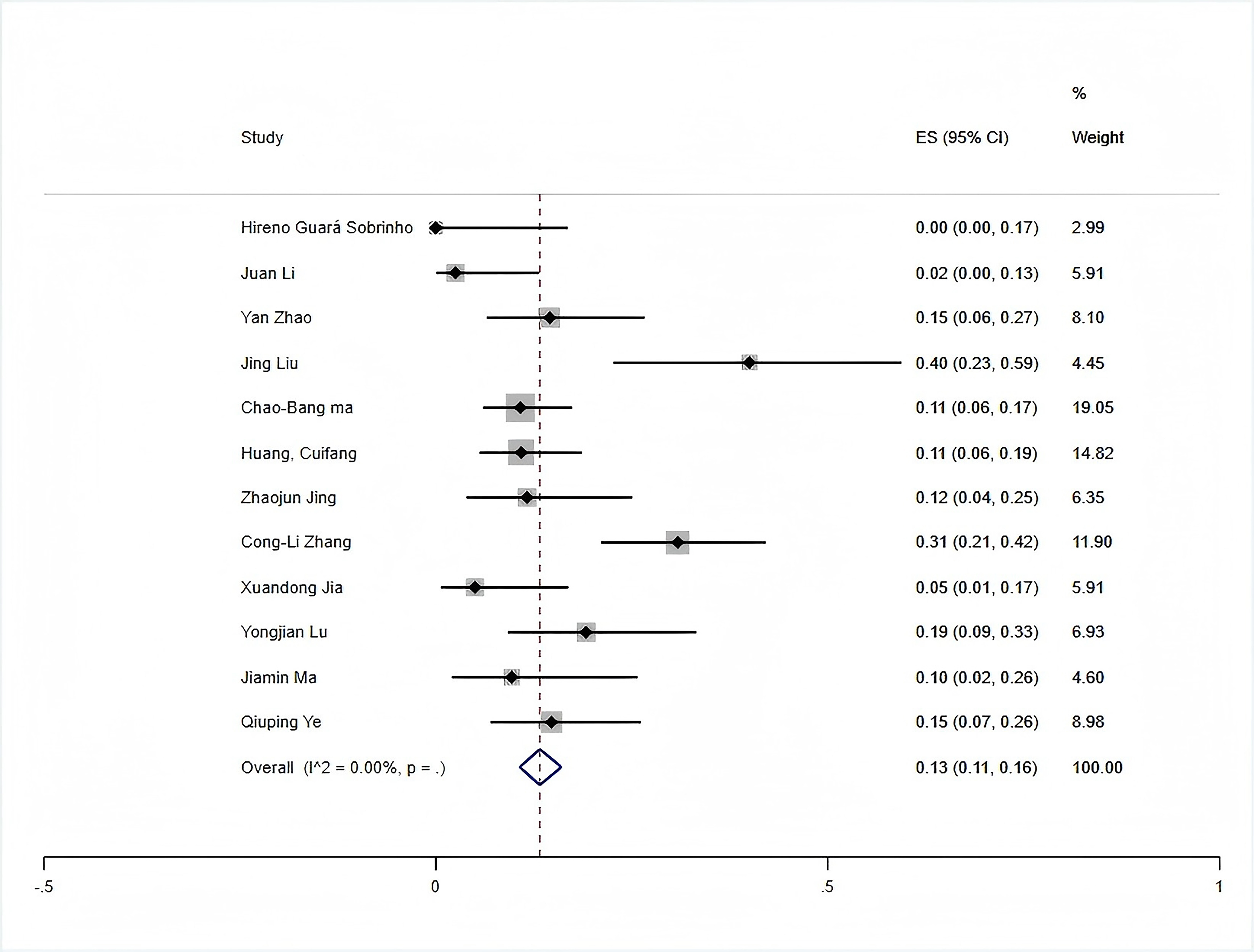

Supplement: Supplementary file 5 [file Image_4.jpeg]
